# Molecular and functional profiling of chemotolerant cells unveils nucleoside metabolism-dependent vulnerabilities in medulloblastoma

**DOI:** 10.1186/s40478-023-01679-7

**Published:** 2023-11-17

**Authors:** Elena Mariotto, Elena Rampazzo, Roberta Bortolozzi, Fatlum Rruga, Ilaria Zeni, Lorenzo Manfreda, Chiara Marchioro, Martina Canton, Alice Cani, Ruben Magni, Alessandra Luchini, Silvia Bresolin, Giampietro Viola, Luca Persano

**Affiliations:** 1 Department of Women’s and Children’s Health, University of Padova, Via Giustiniani 3, 35128 Padua, Italy; 2 Pediatric Research Institute, Corso Stati Uniti 4, 35127 Padua, Italy; 3 Unit of Biostatistics, Department of Cardiac, Thoracic and Vascular Sciences and Public Health, University of Padova, Via Loredan 18, 35131 Padua, Italy; 4 Section of Pharmacology, Department of Pharmaceutical and Pharmacological Sciences, University of Padova, Largo Meneghetti 2, 35131 Padua, Italy; 5 Center for Applied Proteomics and Molecular Medicine, George Mason University, 10920 George Mason Circle, MSN 1A9, Manassas, VA 20110 USA

**Keywords:** Chemotherapy resistance, Medulloblastoma, High throughput drug screening, Antimetabolites

## Abstract

Chemotherapy resistance is considered one of the main causes of tumor relapse, still challenging researchers for the identification of the molecular mechanisms sustaining its emergence. Here, we setup and characterized chemotherapy-resistant models of Medulloblastoma (MB), one of the most lethal pediatric brain tumors, to uncover targetable vulnerabilities associated to their resistant phenotype. Integration of proteomic, transcriptomic and kinomic data revealed a significant deregulation of several pathways in resistant MB cells, converging to cell metabolism, RNA/protein homeostasis, and immune response, eventually impacting on patient outcome. Moreover, resistant MB cell response to a large library of compounds through a high-throughput screening (HTS), highlighted nucleoside metabolism as a relevant vulnerability of chemotolerant cells, with peculiar antimetabolites demonstrating increased efficacy against them and even synergism with conventional chemotherapeutics. Our results suggest that drug-resistant cells significantly rewire multiple cellular processes, allowing their adaptation to a chemotoxic environment, nevertheless exposing alternative actionable susceptibilities for their specific targeting.

**Supplementary Information:**

The online version contains supplementary material available at 10.1186/s40478-023-01679-7.

## Introduction

Despite successful advancements have been made for the treatment and care of patients suffering from several types of cancers, the eventual development of anti-cancer drug resistance still represents a crucial impediment to patient cure. Indeed, the inability to achieve a complete clearance of malignant cells, inevitably affects patient prognosis and outcome [1]. This becomes particularly relevant in the context of pediatric brain tumors, where the therapeutic options are already limited by the tumor localization, unacceptable toxicity of treatments administered in the pediatric age, and high invasiveness of non-pharmacological approaches [2–4].

Although the most widely accepted mechanisms explaining development of resistance are based on the acquisition of genetic alterations affecting drug targets [5, 6], most recent findings suggest the potential involvement of several and even highly different factors dependent on non-mutational mechanisms. These include the enhancement of drug efflux [5–7], induction of adaptive stress responses as lately observed in bacterial antibiotic persistence [8], and chromatin structure plasticity [9, 10]. This complex scenario highlights that potentially hidden mechanisms sustaining chemotherapy resistance still need to be uncovered, thus stimulating the research in the field, with the aim of identifying peculiar resistance-inducing alterations that will serve as promising actionable vulnerabilities for cancer cell eradication.

Here, we generated, characterized, and pharmacologically screened (through an ad hoc HTS approach) chemotherapy-resistant models of medulloblastoma (MB), the leading cause of cancer-dependent deaths in children [11]. Indeed, MBs display rapid growth and highly invasive properties, with 15–30% of them evading/resisting treatments and developing a fatal recurrence [12]. Nonetheless, the highly aggressive treatment schedule, comprising surgical intervention, radiotherapy, and intensive chemotherapy [12, 13], frequently results in a high burden of long-term morbidity due to therapy-induced toxicity [14]. In this context, despite the substantial efforts spent for the identification and characterization of the molecular basis of MB [15], reduction of relapse incidence and improvement of patient survival and quality of life still remain imperative goals to achieve. As a potential explanation, most of current research in MB is based on testing tumor specimens at diagnosis before exposure to any anti-cancer treatment. On the other hand, common pediatric oncology trials involve patients relapsed after treatments, assuming that recurrent tumors should be highly similar to the tumor at diagnosis, even if existing data suggest that potentially actionable targets in the primary tumors are often absent at relapse [16]. This dealignment may severely affect the possibility to identify effective regimens. In addition, since recurrent MB patients do not generally undergo surgery [17], MB studies on relapsed tumors are limited to small sample collections, with poor translational impact. For all these reasons, relevant models of MB chemotherapy resistance may provide invaluable information on the molecular mechanisms sustaining treatment evasion, eventually serving as powerful tools for the identification of alternative therapeutic approaches able to efficiently prevent/target MB drug resistance, with clear expected benefits for patients.

## Materials and methods

### Cell cultures

DAOY (group SHH), HD-MB03 (group 3), and adult normal primary dermal fibroblast cell lines were purchased from ATCC (Manassas, VA). HuTuP33 primary cultures (group 4) were derived from a pediatric MB tumor taken at surgery and were already reported in our previous studies [18].

Med-411 MB cells (group 3) were purchased from the Brain Tumor Resource Laboratory of the Fred Hutchinson Cancer Research Center (“FHCRC”) (Seattle, WA) [19]. Detailed culturing conditions of MB cells are included as Additional file 1: Supplementary Materials and Methods.

### Generation and maintenance of chemotherapy-resistant cells

To generate chemotherapy-resistant counterparts of DAOY, HD-MB03, HuTuP33, and Med-411 cells, they were weekly exposed to a combination of chemotherapeutic drugs (VECC) commonly used during MB patient treatment, including Vincristine (Tocris Bioscience, Bristol, UK), Etoposide (Sigma–Aldrich, St. Louis, MO), Cisplatin, and Cyclophosphamide (both from Santa Cruz Biotechnology, Santa Cruz, CA) [2]. One VECC unit (U) has been defined as the combination of the Growth Inhibitory concentration 50 (GI_50_) of each of the four drugs composing the cocktail, calculated through resazurin (7-Hydroxy-3H-phenoxazin-3-one-10-oxide; Sigma Aldrich, St. Louis, MO)-based dose–response viability assays performed in the DAOY cell line (Additional file 1: Fig. S1A, B).

Resistance was usually acquired by MB cells after 6–9 VECC treatment cycles and further maintained through a weekly 24 h exposure to VECC at cell type-dependent concentrations ranging from 0.1U for DAOY and HuTuP33 cells to 0.2U for HD-MB03 and Med-411 cells.

### Label-free mass spectrometry and data analysis

The proteomic profile of MB-S (Medulloblastoma Sensitive) and MB-R (Medulloblastoma Resistant) cell lines were analyzed by label-free mass spectrometry using a Orbitrap Fusion™ Tribrid™ Mass Spectrometer (Thermo Fisher Scientific, Waltham, MA). More details on sample preparation and raw file data analysis are provided in Additional file 1: Supplementary Materials and Methods and Additional file 4: Table S3.

Differential expressed proteins (DEPs) between MB-S and MB-R cells were identified according to Z score calculated on the median values (Paired *t*-test *p* < 0.05) within the subset of the proteins in common among all the cell models considered (Additional file 2: Table S1). Significant DEPs were clustered using STRING k-means clustering tool (minimum required interaction score: 0.4) and the four resulted clusters (Additional file 2: Table S1) analyzed using the STRING enrichment tool through the Cytoscape platform (v3.9.1).

### Gene expression profiling and data analysis

Total RNA was extracted from MB-S/R cells using QIAzol reagent (Qiagen, Hilden, Germany) according to manufacturer's instructions. For microarray experiments, in vitro transcription, hybridization, and biotin labeling of RNA were performed according to the WT GeneChip Clariom™ S assay (Affymetrix, Santa Clara, CA). CEL files were generated using default Affymetrix microarray analysis parameters (Command Console Suite Software). Expression data were deposited into the Gene Expression Omnibus (GEO) database under Series Accession Number GSE220543 and are accessible without restrictions.

The expression values of differentially expressed genes (DEGs) from MB-R cells (DAOY, HD-MB03, and HuTuP33; N = 12) were correlated to publicly available MB patient transcriptional data (N = 763) from the GSE85217 dataset [20] by using the Perseus software (v1.6.8.0). Spearman r correlation values were used to cluster, by k-means (Euclidean distance), MB patients into 3 main subgroups displaying a low (Cluster 2, n = 260), intermediate (Cluster 1, n = 316), or high (Cluster 3, n = 172) transcriptional similarity with our MB-R cellular models. The Elbow method was used to find the optimal number of clusters for k-means clustering using the common “factoextra” and “cluster” R packages. The identified MB patient clusters were then compared for their overall survival through generation of Kaplan-Meyer curves and Log-rank (Mantel-Cox) statistical analysis.

### Kinase activation analysis

Kinase activation status was assessed using the PamStation®12 (PamGene International,’s-Hertogenbosch, The Netherlands), in MB-S/R cells from DAOY, HD-MB03 and HuTuP33 cell lines through both protein tyrosine kinase (PTK) and serine-threonine kinases (STK) PamChip®4 porous 3D microarrays. Kinase Pathway enrichment analysis was performed with GSEA (C2cp MSigDB) on combined inferred PTK/STK and differentially phosphorylated peptides using Uniprot IDs and their relative PamChip log fold changes.

### High throughput screening (HTS), drug synergism and data analysis

The HTS drug library consisted of 3533 compounds acquired from different commercial sources, including the Prestwick Chemical Library®, a collection of 1280 off-patent FDA-approved drugs (Prestwick Chemical Libraries, Illkirch, France), the DiscoveryProbe™ G Protein-coupled receptor (GPCR) Compound Library and DiscoveryProbe™ Tyrosine Kinase Inhibitor (TKI) Library (both from ApexBio Technology LLC, Houston, TX), in addition to small molecules synthesized by medicinal chemistry academic collaborators. The library composition is summarized in Additional file 1: Fig. S6A and grouped according to their major molecular target or their therapeutic class. Each plate of the screening library contained 320 test compounds while internal negative (DMSO) and positive (Bortezomib) controls were arranged in the external columns 1, 2, 23, and 24 (Additional file 1: Fig. S6B).

To ensure treatment reproducibility, all procedures, including cell seeding, drug dilutions, cell treatment and application of resazurin solution, were carried out through a 96-channel robotic liquid handler (Microlab STAR 96-CORE, Hamilton, Bonaduz, Switzerland).

The day after seeding, cells were treated with the compound library at the final concentration of 5 µM. After 72 h of treatment, a resazurin assay was performed to assess cell viability.

HTS data processing was performed using *cellHTS2* R package [21].

Detailed HTS procedures and data processing are available in Additional file 1: Supplementary Materials and Methods.

### Additional statistical analyses

Graphs and associated statistical analyses were generated using Graph Pad Prism 8.0.1 (GraphPad, La Jolla, CA). All data in bar graphs are presented as mean ± standard error of the mean (S.E.M.). Statistical significance was measured by paired *t*-test. *p < 0.05, **p < 0.01, ***p < 0.001, ****p < 0.0001.

Heatmaps and levelplots displayed in figures have been generated through the Morpheus open access platform (https://software.broadinstitute.org/morpheus).

All HTS data were processed using R 4.2.1 and RStudio Version 2022.07.1.

Additional or more detailed procedures are provided as Additional file 1: Supplementary Materials and Methods.

## Results

### Generation and functional characterization of chemotherapy-resistant cells

**Fig. 1:**
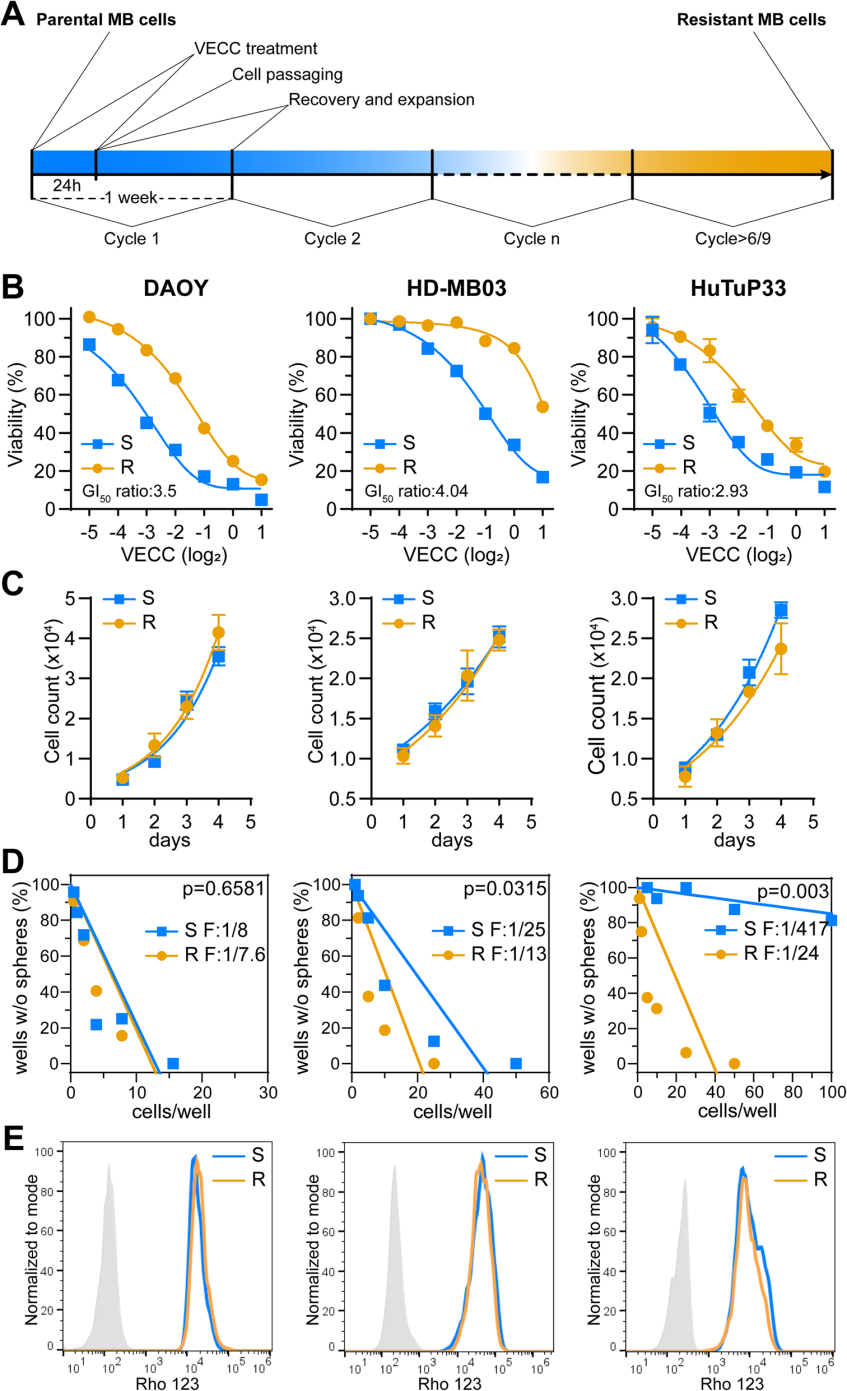
Functional characterization of chemotherapy resistant MB-R cells. **A** Drawing summarizing the VECC treatment schedule (one VECC exposure per week for 24 h) to which MB cell lines and primary cultures (MB-S) have been subjected in order to eventually acquire drug resistant traits (MB-R). **B** Dose–response curves displaying increased resistance to VECC treatment (72 h) of MB-R models relative to their naïve/sensitive counterparts (MB-S). GI_50_ ratio: MB-R GI_50_/MB-S GI_50_ (n = 6). **C** Growth curves showing that both MB-S and MB-R cells possess equivalent proliferation (within 96 h) as measured by trypan blue exclusion assays (n = 3). **D** Limiting dilution assays comparing self-renewal potential of MB-S and R cells. Initiating cell frequency F of cells is reported. p calculated by extra sum-of-squares F test (n = 4). **E** Representative cytofluorimetric plots displaying functional assessment of MB-S/R drug extrusion potential by staining with Rho 123. Control unstained cells are reported as solid grey distributions

Due to the lack of suitable models for studying the mechanisms of drug resistance in MB, we setup chemotherapy tolerant MB models (MB Resistant, MB-R) by weekly treating (see Materials and Methods for more details on treatment schedule) naïve/sensitive MB (MB-S) cell lines (DAOY and HD-MB03) and primary cultures (HuTuP33) with a drug cocktail consisting of four of the most commonly used chemotherapeutics for MB patient therapy [2], including Vincristine, Etoposide, Cisplatin and Cyclophosphamide, namely VECC (Fig. 1A and Additional file 1: Fig. S1A, B). VECC cycles-exposed cells were routinely tested until acquisition of drug resistant traits (commonly after 6–9 treatment cycles) through a resazurin-based cell viability assay. MB-R cells displayed a significant greater resistance to a 72 h VECC exposition (~ 3 to 4 folds in terms of GI_50_ shift) when compared to their relative MB-S counterparts (Fig. 1B), nevertheless showing no evident morphological changes associated to the cycling treatment schedule (Additional file 1: Fig. S1C). Once established and validated, MB-R cells were then functionally characterized and, considering the relative selectivity of most chemotherapeutics toward highly dividing cells, primarily tested for their proliferative potential. To this end, MB cell proliferation was examined through both trypan blue exclusion and EdU incorporation assays which concordantly disclosed identical growth rates to MB-S cells, demonstrating that the observed reduced VECC response was not dependent on differential proliferation (Fig. 1C and Additional file 1: Fig. S1D, E).

According to the “cancer stem cells (CSC) paradigm” and the knowledge that they may display refractoriness to anti-cancer agents by a potential enhanced drug efflux capacity [7], we analyzed both MB-S and MB-R cells for their self-renewing potential through limiting dilution assays. Indeed, HD-MB03-R and HuTuP33-R cells were characterized by a significant increase of self-renewal, with DAOY-R cells displaying no deviations from MB-S cells, probably due to a remarkably high “initiating cell” frequency even in basal conditions (Fig. 1D). However, this increased CSC phenotype was not accompanied by a concomitant enhancement of MB-R cell drug extrusion potential, as verified by a comparable functional efflux of Rhodamine 123, a shared substrate of several multidrug resistance proteins (Fig. 1E and Additional file 1: Fig. S1F). Collectively, these results suggest that additional mechanisms requiring a differential regulation of multiple intracellular pathways, may be involved in sustaining MB-R chemotolerance.

### Proteomic and transcriptomic profiles converge to the identification of relevant pathways fueling MB-R resistance

Biological processes, in both healthy cells and disease, involve a highly dynamic and interactive network of molecular players, including transcripts, proteins, and metabolites, which participate at different levels in defining cell phenotype and behavior. Based on our initial results which preliminarily excluded that the observed MB-R chemoresistance could be dependent on the most common non-mutational mechanisms [6], we hypothesized that treatment resistance in MB would rely on a complex coordination of several biological processes. For this reason, we implemented a multilevel approach based on the integration of both the proteomic and transcriptomic profiles derived from all MB-S/R cells considered in this study.

**Fig. 2:**
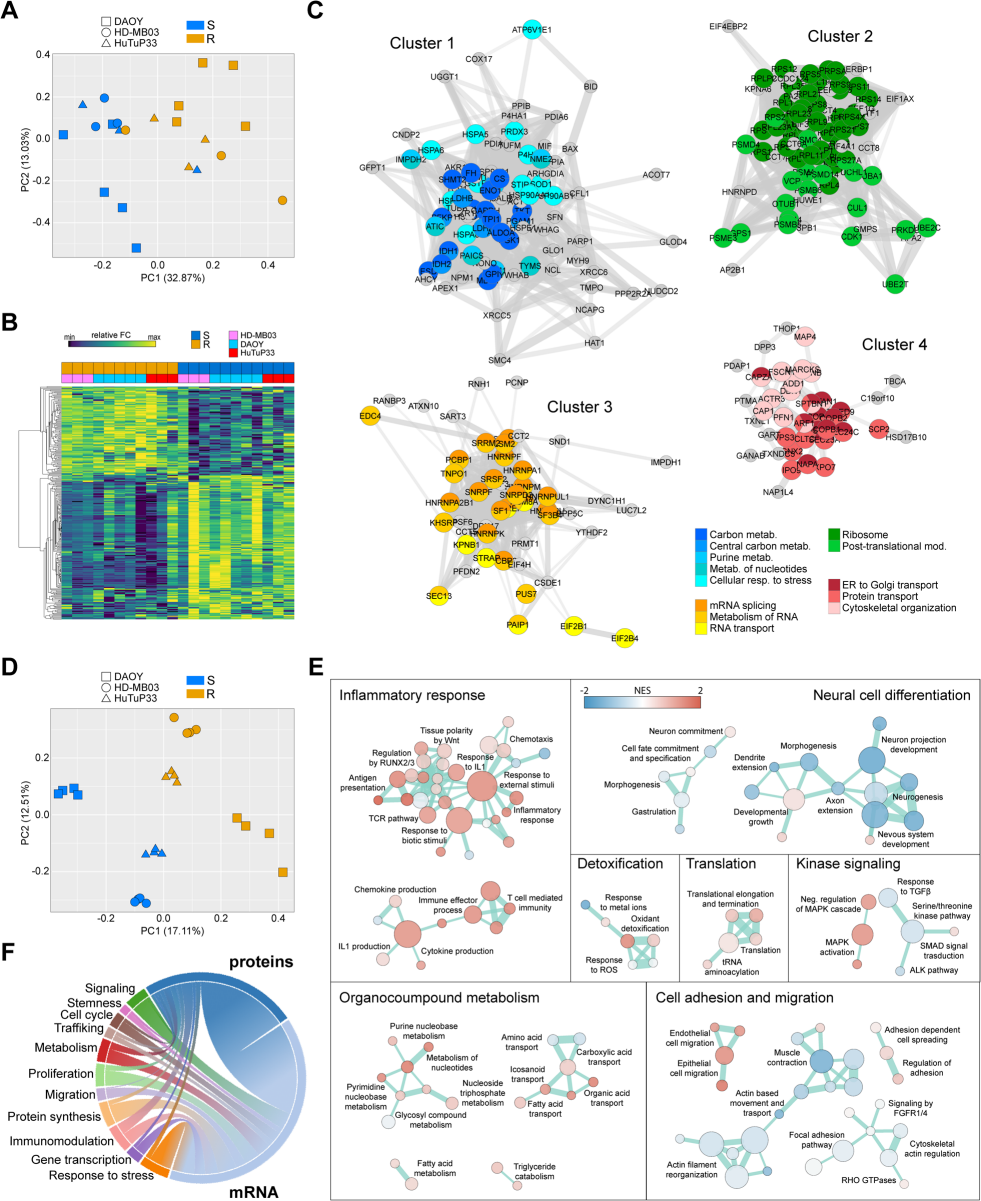
Proteomic and transcriptomic characterization of MB-R/S models. **A** PCA displaying distribution of MB-S/R samples from DAOY (■, n = 5) and HD-MB03 (●, n = 3) cell lines, and HuTuP33 (▲, n = 3) primary cells based on label-free MS-based protein expression data. **B** Heatmap reporting normalized protein abundancy of differentially expressed proteins (DEP, *p* < 0.05, n = 268 by *t* test) between MB-S and MB-R samples as in **A**; FC: fold change. **C** Pathway enrichment analysis (within the Gene Ontology (GO) terms) of DEP clustered into four distinct networks identified through STRING protein–protein interaction analysis (see also Additional file 1: Fig. S3C). Proteins significantly contributing to the reported GO enrichments are highlighted by colored dots. Cluster 1 (shades of blue): n = 86, Cluster 2 (shades of green): n = 85, Cluster 3 (shades of yellow): n = 51 and Cluster 4 (shades of red): n = 39 (Supplementary Table S1). **D** PCA displaying distribution of MB-S/R samples from DAOY (■, n = 4) and HD-MB03 (●, n = 4) cell lines, and HuTuP33 (▲, n = 4) primary cells based on Clariom S™ arrays-based whole transcriptome data. **E** Enrichment map summarizing GSEA results performed in the Hallmarks, C2cp, and C5bp MsigDB gene set collections (q < 0.1 and *p* < 0.05); NES: Normalized Enrichment Score. **F** Chord plot highlighting a high concordance between the enriched cellular processes/pathways identified through proteomic and transcriptomic analyses

Starting from proteomics, matched MB-S/R cells were analyzed through a label-free LC–MS/MS approach, identifying, on average, 1409 ± 370 proteins per sample (for a total of 3506 proteins across all the analyzed MS runs) with a consistent protein expression across biological replicates (Additional file 1: Fig. S2A, B). Moreover, Principal Component Analysis (PCA) disclosed an evident differential distribution of MB-R cell samples relative to their sensitive counterparts (Fig. 2A). Accordingly, we identified 268 shared proteins (Differentially Expressed Proteins, DEPs) displaying a significant differential abundancy, based on their label-free quantification (LFQs) values, between MB-S and MB-R cells (Fig. 2B and Additional file 2: Table S1). With the aim to uncover any potential differentially regulated pathway, we performed String analysis on significant DEPs that allowed their categorization into four major protein–protein interaction clusters through k-means String Network clustering algorithm (Additional file 1: Fig S2C and Additional file 2: Table S1). Pathway enrichment analysis (Fig. 2C) using KEGG and Reactome databases then revealed that chemotherapy resistant cells share an altered expression of proteins involved in multiple processes including: central carbon and nucleoside metabolism (Cluster 1), protein synthesis and post-translational modifications (Cluster 2), mRNA metabolism and transport (Cluster 3), and cytoskeletal organization and intracellular trafficking (Cluster 4), thus suggesting a multi-faced regulation of pro-survival processes upon acquisition of resistant traits in MB cells.

In order to integrate these results into a more comprehensive intracellular network, a similar set of samples from MB-S/R cells was subjected to whole transcriptome analysis through Clariom™ S Affymetrix arrays. This analysis confirmed a clear-cut differential transcriptional behavior across MB-S and R samples (Fig. 2D) and identified 962 differentially expressed genes (DEGs) in MB-R relative to MB-S cells (445 up- and 517 down-regulated; Additional file 3: Table S2). This result corroborates the hypothesis that the acquisition of drug-resistance may be sustained, at least in part, by a peculiar transcriptional shift occurring in MB cells once they are exposed to a recurring chemotherapeutic stimulus. Mirroring the above-described approach of proteomics-driven interpretation of drug resistance, we performed a Gene Set Enrichment Analysis (GSEA) comparing MB-R with MB-S in each cell type. Common enriched pathways from the Hallmarks, C2cp and C5bp MSig databases highlighted a MB-R associated transcriptional dysregulation of several metabolic processes, comprising an increase of nucleoside and fatty acid metabolisms, protein translation, inflammation and immune system-related signaling, and cytoskeleton-dependent processes, including intracellular trafficking and cell adhesion/migration (Fig. 2E). Moreover, MB-R cells were characterized by a strong downregulation of neural differentiation genes (Fig. 2E), in accordance with the increased self-renewal of MB-R cells, already reported in Fig. 1D. Trying to integrate proteomic and transcriptomic information, a comparative analysis demonstrated a dramatic convergence between the identified altered pathways, that portrays MB resistant cells as endowed with a strong dysregulation of several metabolic functions (from biosynthetic pathways to cytoskeleton-dependent processes), remodeled gene transcription and protein translation activities, and increased activation of pro-inflammatory and stress responses (Fig. 2F).

Finally, in order to verify if the observed proteomic and transcriptional changes could be dependent on any recurrent rough genomic aberration accumulating as a consequence of the VECC-dependent DNA-damaging environment, we investigated if genomic copy number evolution (CNE) had occurred in MB-R cells. Indeed, CNE arising in cancer cells during treatments has been widely reported and peculiar therapy-selected cellular clones already recognized in several recent studies [22–25]. Nevertheless, through a methylation array-based identification of MB-S/R DNA copy number alterations, we did not retrieve any recurrent VECC-induced chromosomal aberration across the MB-R models analyzed. In particular, we could detect some additionally deleted regions in DAOY-R chromosomes 2 and a restricted 15q deletion in HuTuP33-R cells (Additional file 1: Fig. S3A, C). Conversely, HD-MB03-R were characterized by a negative selection of peculiar clones bearing discrete 2q and 6q deletions (Additional file 1: Fig. S3B). However, the “classical” pediatric brain tumors-associated genomic alterations (such as *MYC* amplifications or *TP53* deletions [20, 26]) were maintained in MB-R cells and we could not identify any evident correlation between retrieved CNEs and dysregulated genes (not shown). These observations suggest that acquisition of drug resistance and the concomitant observed transcriptional shift is not dependent (or very partially) on CNE in our MB-R models.

### MB patients correlating with a chemotolerant transcriptional phenotype are characterized by worse prognosis

**Fig. 3:**
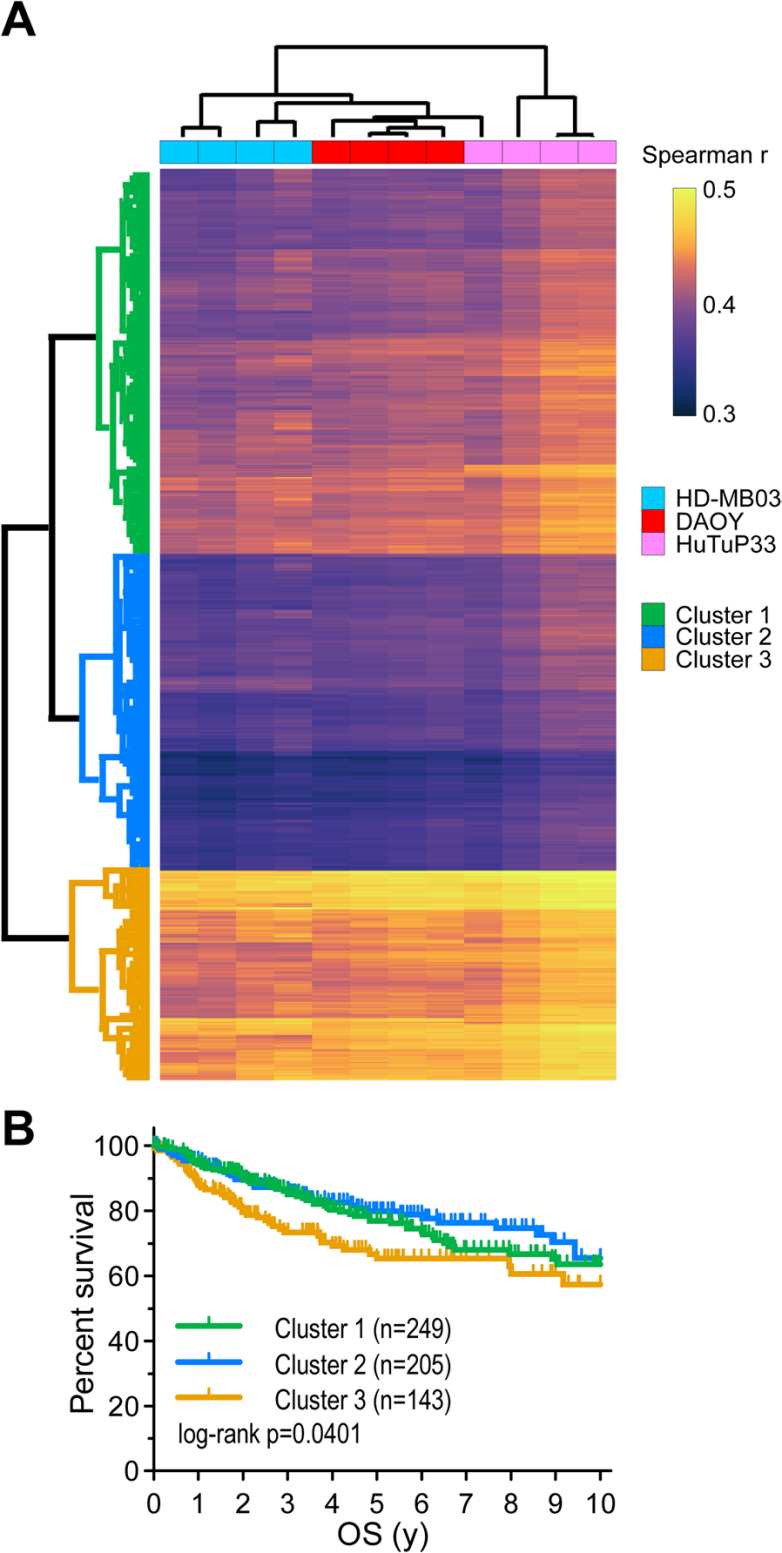
MB patients displaying high correlation with a MB-R associated transcriptional phenotype are characterized by worst prognosis. **A** Heatmap displaying the existing correlation (Spearman r) between MB-R models used within our study and MB patient samples from the GSE85217 dataset [20], based on the expression of previously identified DEGs between MB-S and MB-R models. MB patients have been sub-grouped into 3 different clusters by K means clustering (Euclidean distance). **B** Kaplan Meyer curves comparing the available overall survival (OS) data of MB patients belonging to each Cluster. y: years

Data generated so far, highlight a peculiar rewiring of the transcriptional and proteomic profile of MB-R cells compared to their MB-S fellows and reveal several dysregulated processes potentially contributing to the response to chemotherapy and progressive acquisition of resistance. Based on this information, we wondered if these models could be representative of a subset of highly aggressive and possibly chemotherapy-resistant MB tumors. To verify this hypothesis, we correlated the expression values of the above identified DEGs in MB-R with their corresponding expression levels in tumors belonging to a large pediatric MB patient gene expression cohort (GSE85217) [20]. Interestingly, correlation analysis allowed the identification of three main patient clusters, endowed with a differential grade of correlation with a MB-R dependent chemotolerant transcriptional profile (Fig. 3A). More importantly, MB patients displaying the highest transcriptional correlation with the MB-R chemotolerant state (Cluster 3), were also characterized by a significant worse prognosis in terms of overall survival, if compared to low-correlating tumors (Cluster 2; Fig. 3B). Moreover, when subgrouping MB patients according to their transcriptional subtype classification [15, 26], this differential outcome became even more clear, with Cluster 3 patient displaying a significant more unfavorable outcome in the SHH and Group 4 subgroups (MB patients belonging to the WNT subgroup were necessarily excluded due to their small number and extremely favorable prognosis). Of note, despite not statistically significant, also Cluster 3 patients belonging to Group 3 showed a worsened outcome (Additional file 1: Fig. S4A). These results highlight a strict correlation between our MB-R models and the most aggressive, and possibly therapy resistant, MB tumors retrieved from patients, confirming the relevance of our in vitro setting. Of note, the robustness of these models was supported by the highly consistent data obtained across diverse MB cell lines and primary cultures, and the relative independence from the molecular classification assigned to MB tumors.

### Kinome activation profiling reveals a complex multi-level regulatory network sustaining resistance

**Fig. 4:**
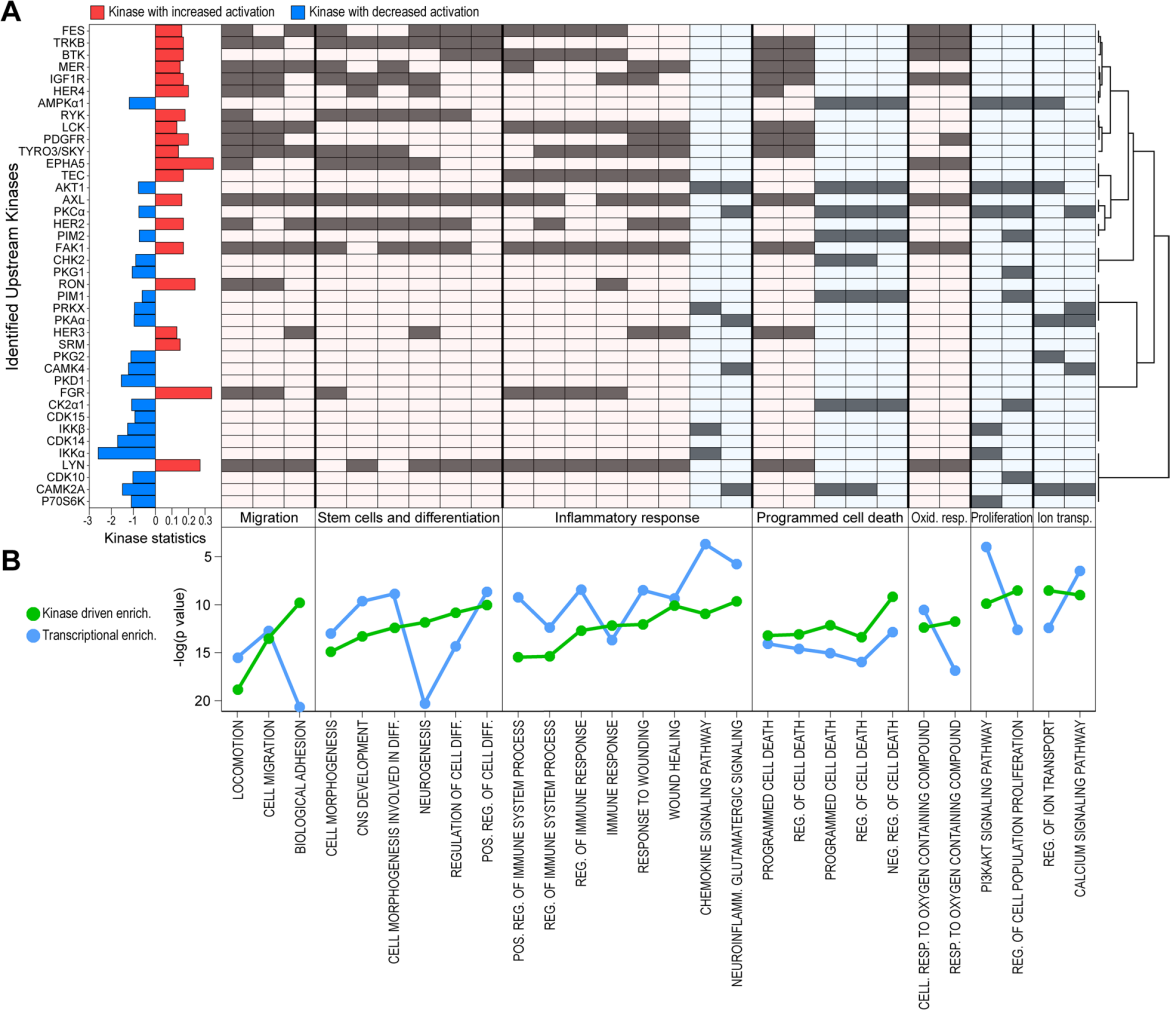
Kinase activation status of MB-S/R cell models. **A** Barplot summarizing the significant differential activation status of indicated kinases (both PTK and STK identified through the PamGene’s UKA) (left panel) and indication of their relative contribution (grey slots; right panel) to gene ontology term enrichments (green lines) reported in **B**. **B** Commonly enriched gene ontology processes between differentially activated kinases (together with their relative differentially phosphorylated peptides; green) reported in **A** and DEGs from gene expression analysis (blue). Positive/negative enrichments are indicated by a differential red/blue coloring of panel sections above

Once verified the applicability of our MB-R models in the characterization of MB resistance to treatments, we evaluated the kinome activation status of MB-R cells and characterized any potential kinase activity deviation relative to sensitive cells. To this end, cell lysates from both MB-S and MB-R cells were subjected to phospho-tyrosine (PTK) and serine-threonine kinome (STK) activity evaluation through the PamGene® technology. Of the 196 PTK and 144 STK phosphorylation sites included into the PamChip® arrays, 17 PTK and 60 STK target peptides resulted as differentially phosphorylated between MB-R and S cells (not shown). According to these data, an upstream kinase analysis-based identification of potential differentially activated kinases in MB-R cells suggested an increased activity of SRC family (FGR, LCK, LYN, SRM), ErbB family (Her2, Her3, Her4), and TAM family (TYRO3, AXL, MER) kinases, and a significant over-activation of PDGFR, IGFR, FAK, Ryk and RON. On the contrary, MB-R cells displayed a reduced activity of kinases involved in calcium signaling (CAMK2A, CAMK4, CK2α1), AMPK/AKT/mTOR axis (PKAα, AMPKα1, AKT1, P70S6K), NF-kB pathway (IKKα and IKKβ), PKGs, and kinases related to cell cycle control including some CDK family members (CDK10, CDK14, CDK15) and CHK2 (Fig. 4A, left panel).

Enrichment analysis based on combined upstream identified kinase activation status and phosphorylation of target peptides was performed through GSEA (Additional file 1: Fig. S5) and integrated with the already available transcriptional enrichments to highlight which relevant pathways, collaboratively affected by both kinase-dependent signaling and transcriptional modulations, were significantly associated with the observed drug resistant phenotype. Overlapping enrichments summarized in Fig. 4B suggest that kinases differentially activated in resistant cells cooperatively affect cell adhesion and migration, control cell differentiation, regulate immune system processes, and enhance the cell response to stress, eventually influencing cell survival. These results further corroborate the hypothesis that the chemotolerant state displayed by MB-R models is sustained, at different levels, by a complex regulatory network involving the concurrent tuning of kinase activation, gene transcription, and protein production.

### High-throughput screening identifies nucleotide metabolism as a functional vulnerability of MB-R cells

The applied multiomic approach defines a significant deregulation of several pathways in resistant MB cells, converging to cell metabolism, RNA/protein homeostasis, and immune response, thus significantly impacting on patient outcome. To identify the specific dysregulated pathways, that functionally sustain the chemotolerant phenotype displayed by MB-R cells, both sensitive and resistant cells were screened for their sensitivity/resistance to a large library of compounds, characterized by widespread targets and mechanisms of action. In particular, the implemented HTS approach consisted in a resazurin-based cell viability screening of 3533 compounds from different commercially available drug libraries, including 1280 FDA-approved drugs (Additional file 1: Fig. S6A). To balance the cohort of our MB-R models, we generated and characterized (according to the same treatment schedule and analytical tools) chemotherapy resistant counterparts of Med-411 primary MB cells, which displayed a consistent behavior with previously described MB-R models in terms of retained morphology, VECC GI_50_ shift, proliferation, self-renewal, drug extrusion capacity, and CNE (Additional file 1: Fig. S7).

**Fig. 5:**
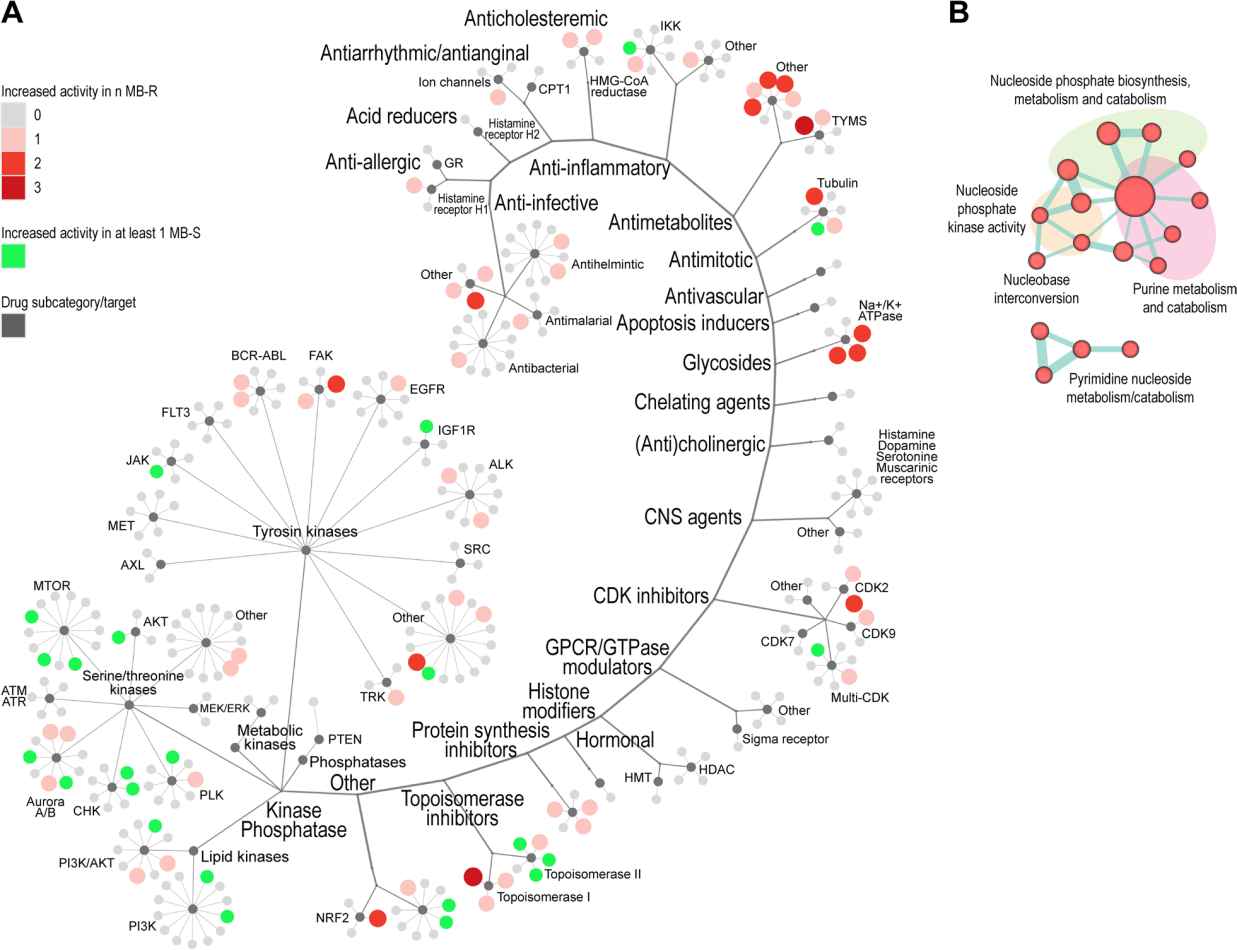
Functional identification of MB-R vulnerabilities by HTS. **A** Distribution of 303 active compounds (5th percentile of all ranked compounds), selected from primary HTS, tested in dose–response experiments, and arranged according to their target/mechanism of action. Each compound is represented as a coloured dot, with increased potency against n MB-R models represented by progressively darker red and increased activity against MB-S cells shown by green color. **B** Enrichment map summarizing transcriptional GSEA results (*p* < 0.05) performed in a subset of Gene Set terms (across the C2cp, and C5bp MsigDB gene set collections) correlated to the antimetabolites function, grouped according to their similarity

A primary screening was performed by treating MB-S/R cells for 72 h at a fixed 5 µM dose for each compound. After data normalization, the robust Z score was used to rank all the tested compounds according to their capacity to inhibit cell growth or cell viability [21]. The top 5th percentile of ranked compounds derived from each screened MB model (cumulatively n = 303) was then additionally tested through 6-points fivefold dilution dose–response experiments in both MB-S and MB-R cells identifying 56 out of 303 compounds (18.48%) with a selective action against one or more MB-R cell models (Fig. 5A, red shaded dots). On the other hand, 23 out of 303 (7.59%) compounds displayed increased activity on at least one MB-S model (Fig. 5A, green dots). Classification of 5^th^ percentile ranked compounds in definite subgroups according to their pharmacological action or target, allowed to easily recognize at least two main drug clusters displaying high selectivity towards MB-R cells, classifying into the “antimetabolites” and “glycosides” compound families. Given the high toxicity displayed by the selected glycosides when administered to normal human cells (Additional file 1: Fig. S8), we excluded this class of drugs and its related biological processes from further analysis. It is well known that most antimetabolites interfere with the metabolic processes responsible for the correct availability of purine or pyrimidine nucleotide precursors, by mildening their synthesis or competing with them during nucleic acid synthesis. Considering that, among the transcriptional changes related to the acquisition of resistant phenotype, different processes associated to the metabolism of nucleosides were specifically enriched (Fig. 2E), we performed an additional GSEA analysis aimed at a more detailed identification of specific enriched gene sets within a collection of antimetabolites-related processes. This confirmed that both purine and pyrimidine metabolic processes are significantly up-regulated in chemotherapy resistant cells (Fig. 5B), suggesting that nucleoside metabolism may represent a potential vulnerability of MB-R cells.

### Antimetabolites sensitize MB-R cells to chemotherapy

**Fig. 6:**
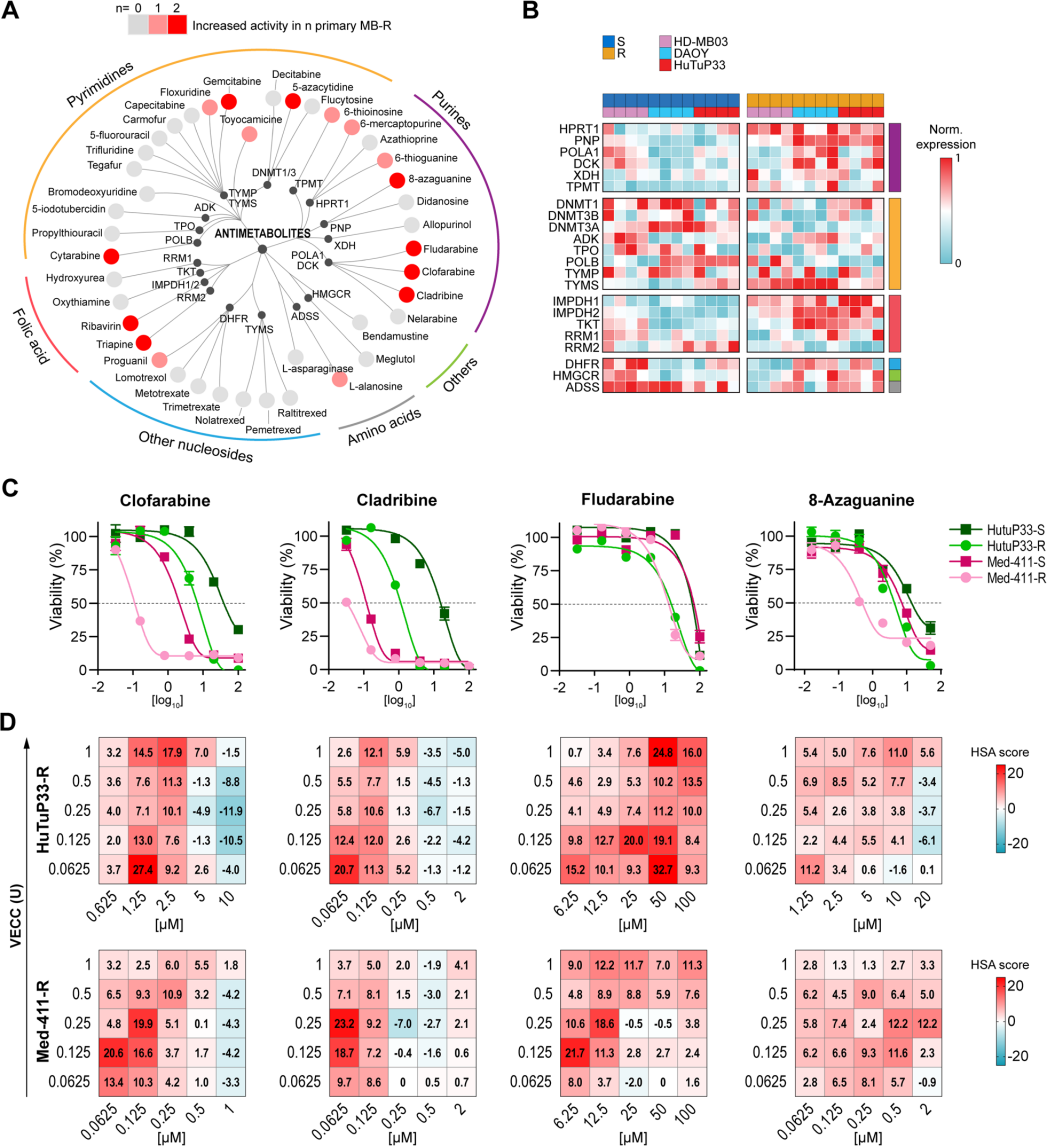
Nucleoside analogues display increased activity in MB-R cells. **A** Distribution of 41 different antimetabolites according to their pharmacological classification and target. Each tested compound is represented as a progressively red dot when displaying increased activity against MB-R primary cultures. **B** Heatmap displaying the differential expression, in MB-R cells, of a subset of genes serving as targets for the antimetabolite drugs tested within the study. **C** Representative dose–response curves of the most active purine analogues against MB-R primary cultures relative to their MB-S counterparts. **D** HSA synergy matrixes calculated for the selected purine analogues as in **C** when combined with VECC in both HuTuP33-R (top panels) and Med-411-R (bottom panels) primary cultures. Compounds have been considered synergistic when HSA ≥ 10, antagonistic when HSA ≤ 10, and non-interactive for 10 > HSA > -10. Relative dose–response matrixes are reported in Supplementary Figure S10

Our experimental approach allowed to functionally select nucleoside metabolism as a relevant cellular process characterizing the resistant phenotype of MB cells which could be then exploited as a specific susceptibility of these cells, with evident therapeutic purposes. Starting from these premises, we expanded the antimetabolite compound collection by including additional molecules possibly excluded from the previous analyses since (i) absent in the HTS library, or (ii) ranked below the 5^th^ percentile. A total of 41 antimetabolites were then tested in dose–response experiments in the two available primary MB-R models. Among all tested compounds, 16/41 (39.02%) demonstrated a significantly increased (GI_50_ ratio > 2) activity against at least one primary MB-R culture (Fig. 6A), thus strengthening the results obtained from the primary HTS. Indeed, by comparing the normalized GI_50_ ratio between MB-S and MB-R pairs, it became particularly clear that the purine analogues class of antimetabolites displayed a remarkable efficacy against MB-R (Fig. 6A and Additional file 1: Fig. S9A). Interestingly, in accordance with the activity of antimetabolites, we found that purine analogues targets were overexpressed in MB-R cells, together with folate metabolism genes, instead of pyrimidine analogues target genes, which rather displayed reduced expression (Fig. 6B). In particular, the adenosine analogues clofarabine (GI_50_ ratio of 5.60 ± 3.38 in HuTuP33 and 18.95 ± 0.07 in Med-411), cladribine (GI_50_ ratio of 18.84 ± 2.44 and 3.54 ± 0.01), and fludarabine (GI_50_ ratio of 3.92 ± 1.84 and 5.27 ± 2.46) and the guanine analogue 8-azaguanine (GI_50_ ratio of 4.22 ± 0.8 and 14.75 ± 3.91) showed a remarkable increased activity against MB-R cells (Fig. 6C and Additional file 1: Fig. S9A, B). As expected, the prodrug nelarabine was inactive in both MB primary cultures, regardless of their resistance status [27]. Of note, almost half of antimetabolites were not toxic in MB cells (within the tested dose range). For these compounds, the GI_50_ values were not calculated due to lack of cell killing/antiproliferative effect (< 50% at the highest dose).

To further increase the potential translational relevance of our functional findings, we conducted drug synergism studies by combining the previously identified most effective purine analogues fludarabine, cladribine, clofarabine, and 8-azaguanine with the VECC chemotherapeutic cocktail in a two-fold dilution matrix layout and evaluated their potential synergism by calculating HSA synergism scores [28]. All tested compounds displayed a significant synergistic action when combined with VECC (HSA score ≥ 10; Fig. 6D and Additional file 1: Fig. S10), suggesting that the combined use of antimetabolites together with the classical MB chemotherapeutics could represent a successful strategy to sensitize MB resistant cells to conventional chemotherapy.

## Discussion

The molecular mechanisms that sustain cancer cell resistance to therapies allowing their adaptation, survival, and re-growth are still poorly understood. Accordingly, the clinical management of recurrent MB tumors is severely affected by inadequate consideration of the high divergence displayed by treatment-evolved MB tumors recurring after therapies [16]. Moreover, the lack of suitable primary-recurrent matched tumor sample collections heavily affects the identification of alternative strategies to prevent relapse or at least effectively treat arising recurrent tumors.

Our experimental approach, allowed to generate and profile relevant in vitro models of chemotherapy-resistant MB cells, evolved after a cycling chemotherapeutic stimulus. Indeed, results provided by this study depict a complex scenario in which several cellular processes adapt to the chemotoxic environment, thus being subjected to a multilevel regulation, from kinase-dependent pathway activation to gene expression. This makes MB cells more refractory to additional chemotherapeutic treatments, but also exposes peculiar susceptibilities depending on the rewiring of definite cellular metabolic functions. In this study, we identified the purine analogues class of antimetabolites as a relevant set of clinically available drugs able, not only to selectively target chemotherapy resistant MB cells, but also to synergize in combination with standard therapeutic regimens. Accordingly, our data provide a first step ahead toward (i) the reduction of high-dose aggressive regimens [2, 29], (ii) the prevention of the long-standing concern regarding therapy-induced neurotoxicity [30, 31] and morbidity [32], and (iii) the setup of more effective treatment options against MB recurrent tumors.

Chemotherapy resistant MB-R models generated within this study are characterized by a prominent increase in their self-renewing capability, suggesting that an increase in the proportion of stem-like cells within the bulk MB cell population could contribute to the acquisition of a chemotolerant state. However, despite transcriptional data suggest that MB-R cells are characterized by a less differentiated phenotype, we could not detect any evident increase in the expression of peculiar CSC protein markers. Of note, CSC markers expression level is already remarkably high in MB-S cells (not shown). Further, the functional and molecular profiling of our resistant MB models excluded the involvement of cellular dormancy, drug efflux, ALDH activity, autophagy, or developmental pathways such as the already reported PI3K/AKT [18], with only a marginal, but not conclusive, increase in their capability to respond to oxidative stress [6, 7, 33].

From a translational point of view, our models were characterized by a molecular phenotype that resulted independent from the genetic and molecular background of each specific cellular model (*MYC* status and/or molecular subgroup), suggesting a shared mechanism engaged by MB cells to survive the chemotoxic environment. This mechanism may represent a crosswise chemotherapy response, with the possibility of translasting a similar approach to other brain tumor contexts, also endowed by molecular heterogeneity. However, despite MB-R cells were characterized by a limited non-recurrent CNE, additional analyses would be needed to rule out potential mutational events responsible for the observed phenotype, as widely reported in several other neoplastic contexts [5, 34–36]. On the other hand, the clear correlation existing between the transcriptional profile characterizing MB-R models and a subgroup of low surviving patients confers clinical relevance to our study and makes us more inclined to sustain the hypothesis of non-mutational events occurring in MB cells during chemotherapy adaptation.

Interestingly, a recent study characterizing the metabolic properties of *MY*C-amplified group 3 MB tumors found out that their genomic context sustains a significant metabolic rewiring, comprising a hyper-activation of de novo nucleoside metabolism and some linked metabolic pathways, together with a collateral shutdown of purine and pyrimidine salvation/degradation processes [37]. Along this line, several recent studies pinpoint that metabolic rewiring must be considered as a fundamental signature of cancer initiation and progression [38–40]. Notably, nucleoside metabolism, in particular the pyrimidine biosynthesis, has been deeply implicated in modulating therapy response of brain tumors [37, 41, 42]. This evidence is unquestionably in line with our data which demonstrate a clear molecular and functional association between increased nucleoside metabolism and a higher sensitivity to purine analogues antimetabolites in chemotherapy resistant MB cells. Moreover, this result highlights that a dysregulation of nucleoside metabolism could be directly involved in sustaining MB chemotolerance. Accordingly, a recent study from Bakhshinyan and colleagues elegantly demonstrated, in a novel PDX mouse-adapted therapy model, that therapy resistant MB cells arising as relapsed tumors were characterized by a prominent dysregulation of cell metabolism at the level of nucleoside (purine) biosynthesis and energy metabolism (OXPHOS and fatty acid) [43]. In line with these results, we functionally identified purine metabolism as a relevant vulnerability of MB cells evolving after chemotherapy and even observed a potential reprogramming of the fatty acids contribution to the energy metabolism as described in other tumors [44, 45], including brain [46]. Moreover, a previous study linked the rewiring of RNA metabolism to the aggressive behavior of group 3 MB tumors [39] and here we show that this could be also considered as an anticipated hallmark of therapy resistance in the MB context.

Pharmacologically, our HTS approach demonstrated that MB-R cells are characterized by a differential response to several compounds, including an increased resistance to certain agents, even anticipated by molecular data. For example, MB-R cells display a general increased resistance to the inhibition of the PI3K/AKT/mTOR axis members, in line with the observed reduced activation of the signaling through kinome analysis. In the same way, the increased resistance to topoisomerase II inhibitors could be easily explained by the use of etoposide within the VECC combination. On the other hand, we could observe a mild increase of MB-R cell sensitivity to certain topoisomerase I inhibitors. The most promising result achieved within this study is the functional validation of nucleoside analogues as relevant sensitizing agents against chemotherapy resistant MB cells. The previous identification, through an HTS approach, of the nucleosides derivatives Gemcitabine and Pemetrexed as highly effective agents against *MYC*-amplified group 3 MB tumors [47] corroborates our results and even further highlights the relevance of our findings.

Clinically, purine analogues are known to cross the blood brain barrier since they could induce side neurotoxic effects when administered at very high doses against extra-cerebral tumors [48]. Nevertheless, fludarabine and cladribine have been widely used for the treatment of indolent lymphoid malignancies and non-Hodgking lymphoma [49], while the second-generation clofarabine has been introduced for pediatric patients with advanced and refractory acute leukemias [50]. The results provided within our study and the clear synergistic action of purine analogues, including clofarabine, when administered, at least in vitro, in combination with other chemotherapic cocktails, dramatically enhance the translational potential of our results and even make the use of these compounds clinically attractive.

In conclusion, the chemotherapy resistant models described in this work fulfill the need for reliable models of recurrent disease in MB, where the lack of relapsed tissues hampers the identification of the molecular players responsible for tumor regrowth. Despite highly promising, our results will need further interpretation in order to unveil additional crucial biological nodes contributing to the observed chemotolerant phenotype.

## Conclusions

Results obtained within our study possess a cutting-edge significance with anticipated impact on the research on MB. Indeed, we unveiled a peculiar rewiring occurring in resistant MB cells, which displays the upregulation of specific pathways possibly involved in fueling resistant cell survival and response to chemotherapeutics. These data greatly increase the knowledge of the molecular events engaged by chemotherapy escaping cells and how these cells can adapt to the chemo-toxic microenvironment. In addition, resistant MB cells possess specific transcriptional features impacting, not only on drug response, but also on MB patient prognosis. More importantly, data produced by the employed HTS approach allowed the identification of purine analogues as efficacious compounds against chemotherapy-resistant MB cells. These results increase the relevance of our findings since identified drugs have been formerly approved for the treatment of other malignancies, with the promise of their easy clinical translation in the context of MB.

### Supplementary Information

**Additional file 1**. Supplementary materials and methods. Supplementary figures S1 to S10.

**Additional file 2**. Supplementary Table S1.

**Additional file 3**. Supplementary Table S2.

**Additional file 4**. Supplementary Table S3.

## Data Availability

The gene expression datasets generated during the current study are available in the GEO repository under Series Accession Number GSE220543 and are accessible without restrictions.

## Abbreviations

CNE: Copy number evolution
CSC: Cancer stem cells
DEG: Differentially expressed genes
DEP: Differentially expressed proteins
GEO: Gene expression omnibus
GI50: Growth inhibition 50
GPCR: G Protein-coupled receptor
GSEA: Gene set enrichment analysis
HSA: Highest single agent
HTS: High throughput screening
LFQ: Label free quantification
MB: Medulloblastoma
MB-R: Medulloblastoma resistant cells
MB-S: Medulloblastoma sensitive cells
OXPHOS: Oxidative phosphorylation
PCA: Principal component analysis
PDX: Patient derived xenograft
PTK: Protein tyrosine kinases
SEM: Standard error of the mean
SHH: Sonic hedgehog
STK: Serine/threonine protein kinase
TKI: Tyrosin kinase inhibitor
VECC: Vincristine, etoposide, cisplatin, cyclophosphamide
