# Supplementary material for: Molecular and functional profiling of chemotolerant cells unveils nucleoside metabolism-dependent vulnerabilities in medulloblastoma
Source: Acta Neuropathol Commun. 2023 Nov 17;11:183. doi: 10.1186/s40478-023-01679-7 (PMC10655385; doi:10.1186/s40478-023-01679-7)
Supplement: Supplementary file 1 — Additional file 1. Supplementary materials and methods. Supplementary figures S1 to S10. [file 40478_2023_1679_MOESM1_ESM.pdf]

Supplementary Information for

Molecular and functional profiling of chemotolerant cells unveils nucleoside metabolism-dependent vulnerabilities in medulloblastoma.

Elena Mariotto, Elena Rampazzo*, Roberta Bortolozzi*, Fatlum Rruga, Ilaria Zeni, Lorenzo Manfreda, Chiara Marchioro, Martina Canton, Alice Cani, Ruben Magni, Alessandra Luchini, Silvia Bresolin, Giampietro Viola, and Luca Persano.

*Corresponding author. Email: elena.rampazzo@unipd.it, roberta.bortolozzi@unipd.it

This PDF file includes:

Supplementary Materials and Methods
Supplementary Figures S1 to S10

Other Supplementary Information for this manuscript include the following:

Supplementary Tables S1 to S3

SUPPLEMENTARY MATERIALS AND METHODS

Cell cultures.

DAOY and HD-MB03 cells were purchased from ATCC (Manassas, VA) and cultured in MEM-ALPHA and RPMI 1640 (Thermo Fisher Scientific, Waltham, MA), respectively, both supplemented with 10% fetal bovine serum (FBS), 1% glutamine and 1% Penicillin-Streptomycin (stock solutions: Penicillin 10000units/mL and Streptomycin 10mg/mL; Thermo Fisher Scientific, Waltham, MA). HuTuP33 primary cultures were derived from a pediatric MB tumor taken at surgery and were already reported in our previous studies.

Med-411 MB cultures were purchased from the Brain Tumor Resource Laboratory of the Fred Hutchinson Cancer Research Center ("FHCRC") (Seattle, WA). Both HuTuP33 and Med-411 primary MB cells were cultured onto fibronectin-coated plates in DMEM/F12 (Biowest, Nuaillé, France) supplemented with 2% B27 (Thermo Fisher Scientific, Waltham, MA), 40ng/ml basic Fibroblast Growth Factor (bFGF) and 40ng/ml Epidermal Growth Factor (EGF; both from Cell Guidance Systems Ltd, Cambridge, UK). Primary MB cultures were maintained in an atmosphere of 2% oxygen, 5% carbon dioxide and balanced nitrogen in a H35 hypoxic cabinet (Don Whitley Scientific Ltd, Shipley, UK) to better resemble the hypoxic conditions of MB tumor microenvironment, ensuring a proper expansion of MB cell precursors21. For continuous expansion, one-half of medium was replaced every two days and cultures passaged every 7-10 days using TrypLE Express (Invitrogen, Carlsbad, CA).

Adult normal primary dermal fibroblasts were purchased from ATCC and cultured in DMEM (Thermo Fisher Scientific, Waltham, MA) supplemented with 10% fetal bovine serum (FBS), 1% glutamine and 1% Penicillin-Streptomycin.

Cultures were passaged for no more than 10 consecutive passages in order to avoid long term culture-related effects.

Brightfield images of cultured cells have been acquired with a Nikon TS100 inverted microscope (Nikon, Melville, NY).

Cytofluorimetric analyses.

5-ethynil-2'-deoxyuridine (EdU) incorporation was performed according to the Baseclick EdU Flow Cytometry Kit instructions (Sigma-Aldrich, St. Louis, MO). In particular, 10µM EdU was incorporated for 16h by both MB-S and MB-R cells. Then, EdU positivity was detected with a Cytomics FC500 flow cytometer (Beckman Coulter, Brea, CA).

Rhodamine 123 (Rho 123; Thermo Scientific Pierce Protein Biology, Waltham, MA) was performed in order to assess MB-S/R drug extrusion capability. Briefly, cells were harvested and 0.1µM Rho 123 was incubated for 15min at room temperature (RT), before being detected through a Cytomics FC500 flow cytometer (Beckman Coulter, Brea, CA). Both EdU and Rho 123 raw data were analyzed with FlowJo v10.8.1 software (BD Bioscience, Franklin Lakes, NJ).

Limiting dilution assays.

To assess their self-renewal, both MB-S and MB-R cells were expanded and then plated in serial dilutions ranging from 500 to 1 cell/well in ultra-low attachment 96-well plates (Corning, Glendale, AZ) using a MoFlo XDP cell sorter (Beckman Coulter, Brea, CA). Cells were then cultured for 10 additional days and then the proportion (%) of wells in which sphere formation was not observed was calculated. Statistical significance was calculated by the extra sum-of-squares F test.

Label-free mass spectrometry and raw data analysis.

MB cells were lysed in TPER lysis buffer (Thermo Fisher Scientific, Waltham, MA) containing Halt protease and phosphatase inhibitors cocktail (Thermo Fisher Scientific, Waltham, MA) and then each sample (50µg of total proteins) denatured and reduced in 6M urea and 10mM dithiothreitol (DTT) for 30min at 37°C. Alkylation was performed with 30mM iodacetamide for 20min at RT. Enzymatic digestion was carried out overnight at 37°C with 2µl (0.5mg/ml stock solution) of sequencing grade trypsin (Promega, Madison, WI) in 50mM ammonium bicarbonate, pH 8.0. Digestion was stopped with 100% trifluoroacetic acid and samples were desalted in Pierce C-18 spin columns (Thermo Fisher Scientific, Waltham, MA). Final eluates were dried using a Microvap 118 nitrogen evaporator (Organomation, Berlin, MA). Samples were reconstituted in 10µl of 0.1% formic acid and analyzed with a Orbitrap Fusion™ Tribrid™ Mass Spectrometer (Thermo Fisher Scientific, Waltham, MA). The mass spectrometer was operated in a data-dependent mode in which each full MS scan was followed by five MS/MS scans where the top five most abundant molecular ions were dynamically selected for collision induced dissociation (CID) using a normalized collision energy of 35%.

Raw files were processed with MaxQuant software (v1.6.10.43) executing the Andromeda search engine fig against the SwissProt human proteome containing 20364 entries (http://www.uniprot.org; updated on March, 2019). Contaminant and reverse decoy databases were enabled, and the standard MaxQuant search parameters were used: tryptic digestion with maximum of 2 missed cleavages; carboimidomethyl (C) as fixed modification; oxidation (M) and Acetyl (N-term) as variable modifications. A complete list of the parameters used in MaxQuant can be found in Supplementary Table S3. Proteins were quantified by Label-free quantification (LFQ) which was carried in MaxQuant using unique peptides and the LFQ min. ratio count set to 1. A complete list of the parameters used in MaxQuant can be found in Supplementary Table S3. Normalization was enabled during LFQ. Post search data analysis was performed using Perseus (v1.6.8.0) [1] and missing values inferred and replaced according to normal distributions.

Gene expression profiling data analysis.

Microarray data (CEL files) were generated using default Affymetrix microarray analysis parameters (Command Console Suite Software). CEL files were normalized using the robust multiarray averaging expression measure of Affy-R package (www.bioconductor.org). Differentially expressed genes (DEGs) between MB-S and MB-R cells (Suppl. Table S2) were identified using Significance Analysis of Microarray (SAM) algorithm coded in the samr R package [2]. From SAM, we estimated the percentage of false positive predictions (False Discovery Rate, FDR) with 100 permutations. Genes with a calculated FDR<0.05 were considered significant. Expression data were deposited into the Gene Expression Omnibus (GEO) database under Series Accession Number GSE220543 and are accessible without restrictions.

Enrichment analysis was performed using Gene Set Enrichment Analysis (GSEA) and enrichment plots were generated through the Enrichment Map application (v3.3.4) of the Cytoscape platform (v3.9.1). In general, FDR<0.1 and Jaccard>0.25 were used as cutoff parameters for significant enrichments and gene set interactions, respectively.

Kinase activation analysis.

To assess kinase activation profile of MB-S and MB-R cells, total cell lysates were prepared using M-PER Mammalian Extraction Buffer supplemented with Halt Phosphatase and Protease inhibitors (all from Thermo Fischer Scientific, Waltham, MA) and then shipped to Pamgene's facilities [3, 4] where they were hybridized to PamChips containing 196 (PTK) or 144 (STK)

reporter peptides (which represent all the possible targets for the active kinases) in order to detect differential intracellular kinase-driven peptide phosphorylations during time between MB-S and MB-R cells (n=4 for each cell model used). Pamchip data were then analyzed using the Pamgene's BioNavigator® software, allowing interpretation, visualization, and computation of the PamChip measurements. Signal ratios were used to calculate the fold change of each phosphopeptide averaged across the analyzed replicates. Differential phosphopeptide signals greater than or equal to 30% have been considered for paired t test analysis ($p<0.05$ cutoff). Pamgene's Upstream Kinase Analysis (U.K.A.) has been used to generate a ranked list of inferred differentially activated kinases.

HTS procedures and data processing workflow.
The stock screening library (10mM in DMSO) and the intermediate dilution (1mM in DMSO) were stored at -80°C in 384-well deep-well plates (#781270 Greiner Bio-One, Kremsmünster, Austria).
All MB cells subjected to HTS were first tested for optimal cell density (data not shown). HD-MB03, HuTuP33, Med-411 cells were seeded at 2000 cells/well, while DAOY were seeded at 400 cells/well in 27µl/well of their complete growth medium in 384-well flat bottom polystyrene plate (#3701 Corning, Corning, NY).
The day after cell seeding, the 1mM stock library was freshly diluted into a 50µM working library in Hank's balanced salt solution without Ca^{2+} and Mg^{2+} (HBSS, Biosera, Nuaille, France) to avoid compound precipitation or inactivation. 3µl of each compound from the working library dilution, were added to cells to achieve a final concentration of 5µM. After 72 hours of treatment, 3µl of resazurin (10x) was added to each well to reach a final concentration of 44µM. After 3-6 hours of incubation, depending on MB cells, the fluorescence signal was measured at 590nm using a multi-well plate reader (Spark, Tecan, Männedorf, Switzerland).
To assess the robustness of our experimental and analytical pipeline, the Z-prime factor (Z') was monitored throughout the entire campaign, allowing the measurement of the separation between positive and negative controls, and only plates with Z'>0.5 were analyzed to ensure trustworthiness of results (Suppl. Fig. S6B-D).

The HTS data processing workflow can be summarized by the following steps:

Plate quality control
The Z-prime (Z') quality control metrics was calculated for each plate throughout the entire HTS to measure the separation between the positive control (200nM Bortezomib) and negative control (0.5% DMSO) after 72 hours of drug exposure. The increasing trend of Z' statistic over the number of screened plates reflected the adoption of the gas-permeable sealing membranes (Breathe-Easy, Merck, Readington Township, NJ) used to avoid evaporation during treatment incubation, reaching overall negligible plate bias with excellent quality metrics (Z' > 0.7), thus indicating HTS robustness and data consistency (Suppl. Fig. S6C). For this screening, only plates with acceptable quality metrics (Z' > 0.4) were further analyzed.

Plate Normalization
To adjust per-plate technical variations, raw data were first normalized by calculating the relative signal of each well compared with the median of all the samples by the following calculation:

$$x'ki = \frac{xki}{X}$$

Where x_{ki} is the raw fluorescence intensity for the kth well in the ith plate, and x'_{kj} is its normalized intensity. X is the median calculated among all sample wells, excluding reference wells containing controls.

Standardization of replicates

The robust Z-score was used to measure outlier strength (i.e. HTS hits) using robust estimators of spread (median and median absolute deviation (MAD) instead of mean and standard deviation) to re-scale normalized data relative to plate-to-plate variation within each replicate. The robust Z-score method excludes control measurements under the assumption that most HTS compounds are inactive and behave as negative controls (DMSO).

Mathematically, the robust Z-score is defined as:

$$z'kj = \frac{x'kj - X'}{MAD}$$

Where x'_{kj} is the normalized value for the kth well in the jth replicate, while X' and MAD are the median and the median absolute deviation of the distribution of all normalized values of the jth replicate, respectively.

Compounds ranking

The median Z-score was finally chosen to rank all tested compounds according to their antitumoral activity for each MB cell line tested.

Second round screening

The top 5th percentile of most active drugs retrieved from first-round screening from all MB-S/R cells were cherry-picked from the 10mM stock library and re-tested through 6-points 5-fold dose-response curves (two technical replicates per plate in two independent experiments). The response of cells to the compounds were normalized according to the following equation:

$$cell\ viability\ (\%) = \frac{\mathrm{x} - \mathrm{POS}}{\mathrm{NEG} - \mathrm{POS}} * 100$$

where x is the relative fluorescence units (RFU) collected from each single well, NEG is the mean of intraplate negative controls (0.5% DMSO), and POS is the mean of intraplate positive controls (200nM Bortezomib). Dose-response curves were fitted using the four-parameter logistic (4PL) regression model using the *GRmetrics* R package [5]. Data are represented as mean ± standard error of the mean (SEM) of replicate experiments and graphed using Graph Pad Prism 8.0.1 (GraphPad, La Jolla, CA). Finally, the MB-R/MB-S GI_{50} ratio was used to determine resistant-selective compounds (Fig. 5A). To further skim the list of resistant-selective compounds, the top 5th percentile of active drugs identified in first-round screening were tested in dose-response experiments also on primary human dermal fibroblasts (300 cells/well) to remove undesired cytotoxic effect (growth inhibition ≥ 25%) on normal cells (Suppl. Fig. S8A). The same approach, including both experimental and analytical procedures) was then implemented in order to screen

the activity of additional reported antimetabolites (all from MedChemExpress, Monmouth Junction, NJ) (Fig. 6A, C and Suppl. Fig. S9).

Drug synergism studies

Briefly, 2000 PD-MB-R primary cells per well were seeded in 384-well plates in 24µl of complete growth medium. After 24h, cells were treated with 3µl of 10x purine analogues (clofarabine, cladribine, fludarabine, or 8-azaguanine) as single treatments or in combination with 3µl of 10x VECC in a 5x5 matrix design (Fig. 6D). Each dose was tested in duplicate within each plate, in two independent experiments. Raw data were normalized as described for second-round screening and then processed using *Synergyfinder* R package [6] to finally compute the Highest Single Agent (HSA) synergy score for all technical replicates per dose per drug combination (Fig. 6D and Suppl. Fig. S10).

Supplementary Figure S1

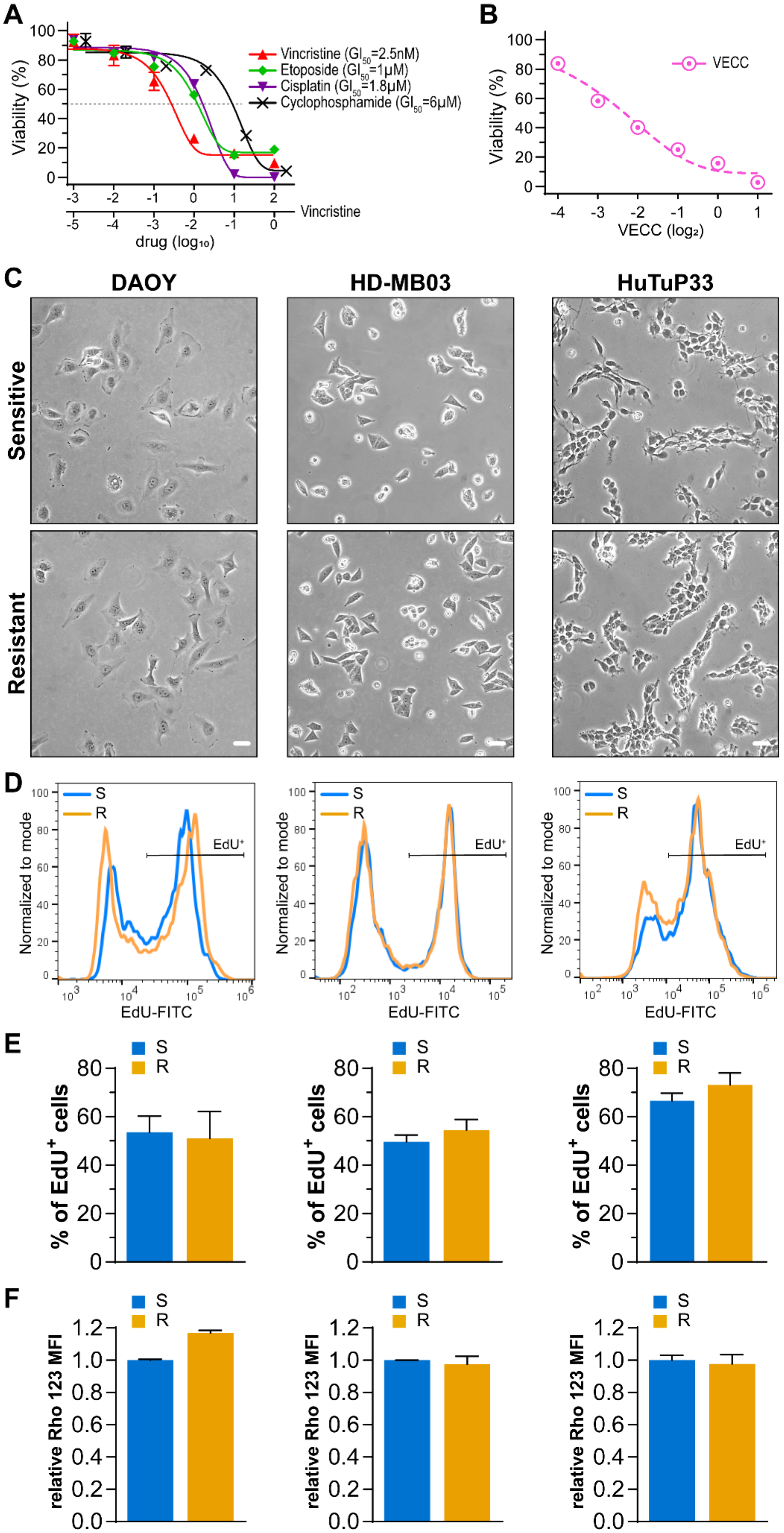

Supplementary Figure S1. Morphological and functional characterization of MB-S and MB-R cellular models. (**A**) Dose-response curves displaying the effects of Vincristine (red), Etoposide (green), Cisplatin (purple), and Cyclophosphamide (black), when administered as single agents for 72h to DAOY-S cells. GI_{50} values are highlighted by curve intersection with the 50% viability dotted line. (**B**) Representative dose-response curve of the VECC cocktail (composed by the GI_{50} concentration of the four drugs as in (**A**)) in DAOY-S cells. (**C**) Representative brightfield images displaying no evident morphological differences between MB-S and MB-R models used within our study. Original magnification 10X; bar: 20µm. (**D**, **E**) Representative cytofluorimetric plots (**D**) and relative quantification (**E**) of MB-S/R cell proliferation as measured by the amount of EdU incorporation within 16h (n=5 independent experiments). (**F**) Relative quantification of drug efflux potential of MB-S/R cells as measured by detection of Rho 123 extrusion by flow cytometry (n=3 independent experiments).

Supplementary Figure S2

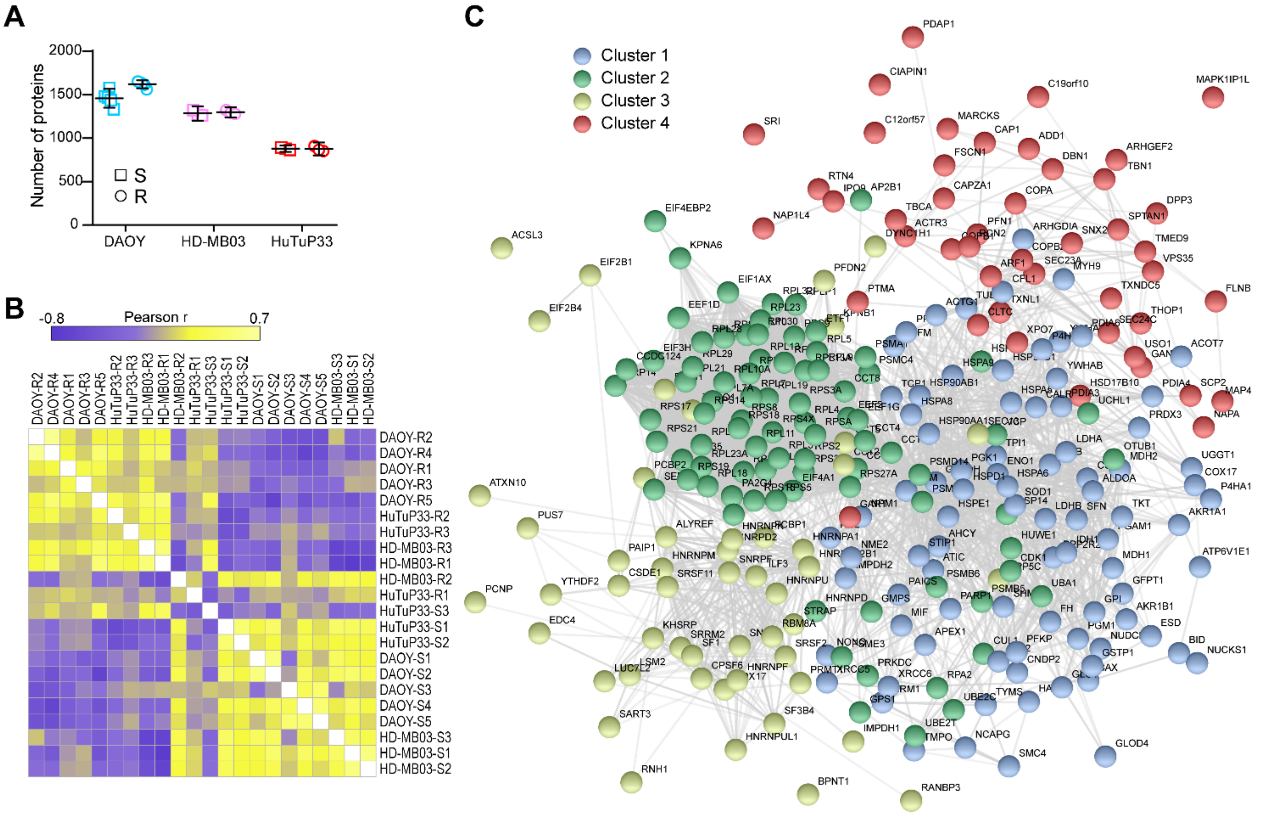

Supplementary Figure S2. Proteomic characterization of MB-S/R cells. (A) Graph reporting the number of unique proteins identified in MB-S/R samples according to the implemented label-free MS approach. (B) Correlation matrix displaying inter-sample correlation (Pearson r) according to identified protein expressions in MB-S and R samples. (C) Comprehensive STRING network analysis displaying interactions occurring between DEPs unveiled from MB-S vs MB-R comparison (Fig. 2B) and identification of four protein clusters based on their GO annotation. The same protein clusters (1-4) have been further characterized for their enrichment in the KEGG pathways (Fig. 2C).

Supplementary Figure S3

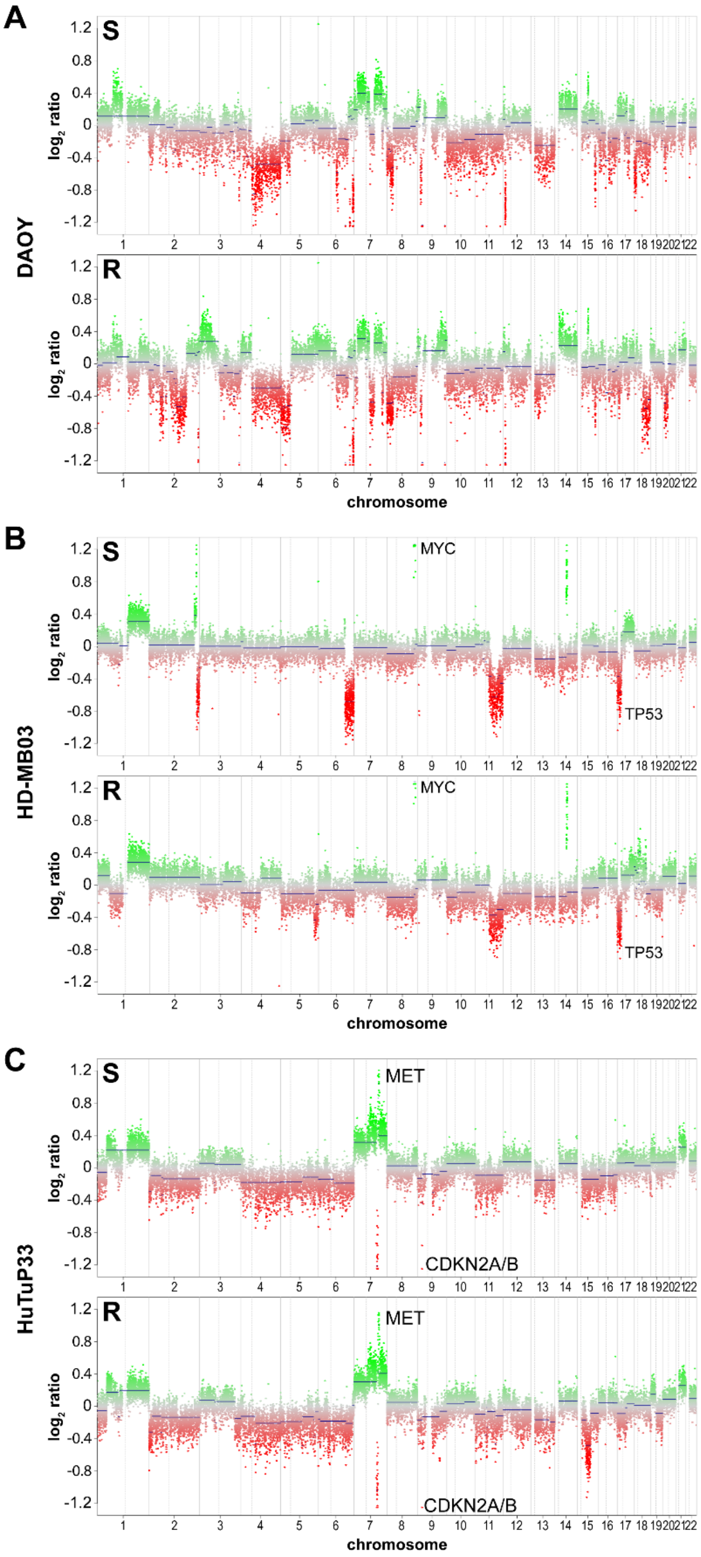

Supplementary Figure S3. Copy number evolution of MB-R cells. Representative genome wide plots (excluding sex chromosomes) displaying positive and negative deviations of the indicated genomic regions of MB-S/R cells from a normalized reference, representative of

gains/amplifications (log2 ratio>0.5, shades of green) or losses (log2 ratio<-0.5, shades of red), respectively, according to Illumina EPIC arrays-based DNA methylation profiles [7].

Supplementary Figure S4

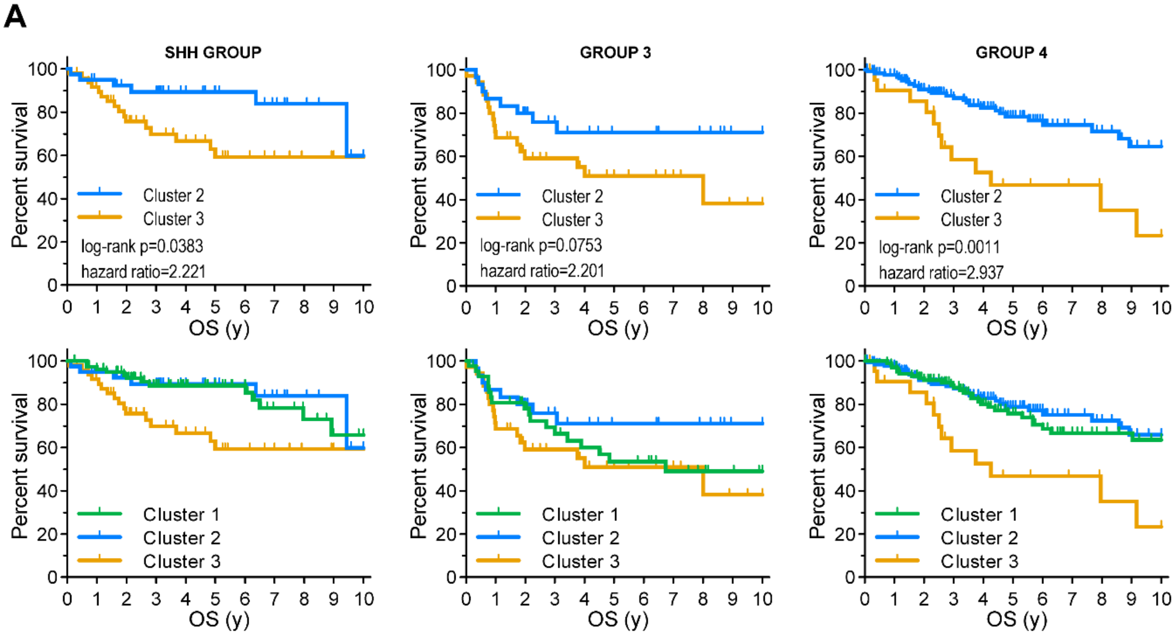

Supplementary Figure S4. Survival of MB patients belonging to different molecular subgroups according to their correlation with MB-R transcriptome-driven clusters. (**A**) Kaplan Meyer survival curves of MB patient clusters identified in Fig. 4A and subsampled according to their previously assigned molecular subgroup [8]. y: years.

Supplementary Figure S5

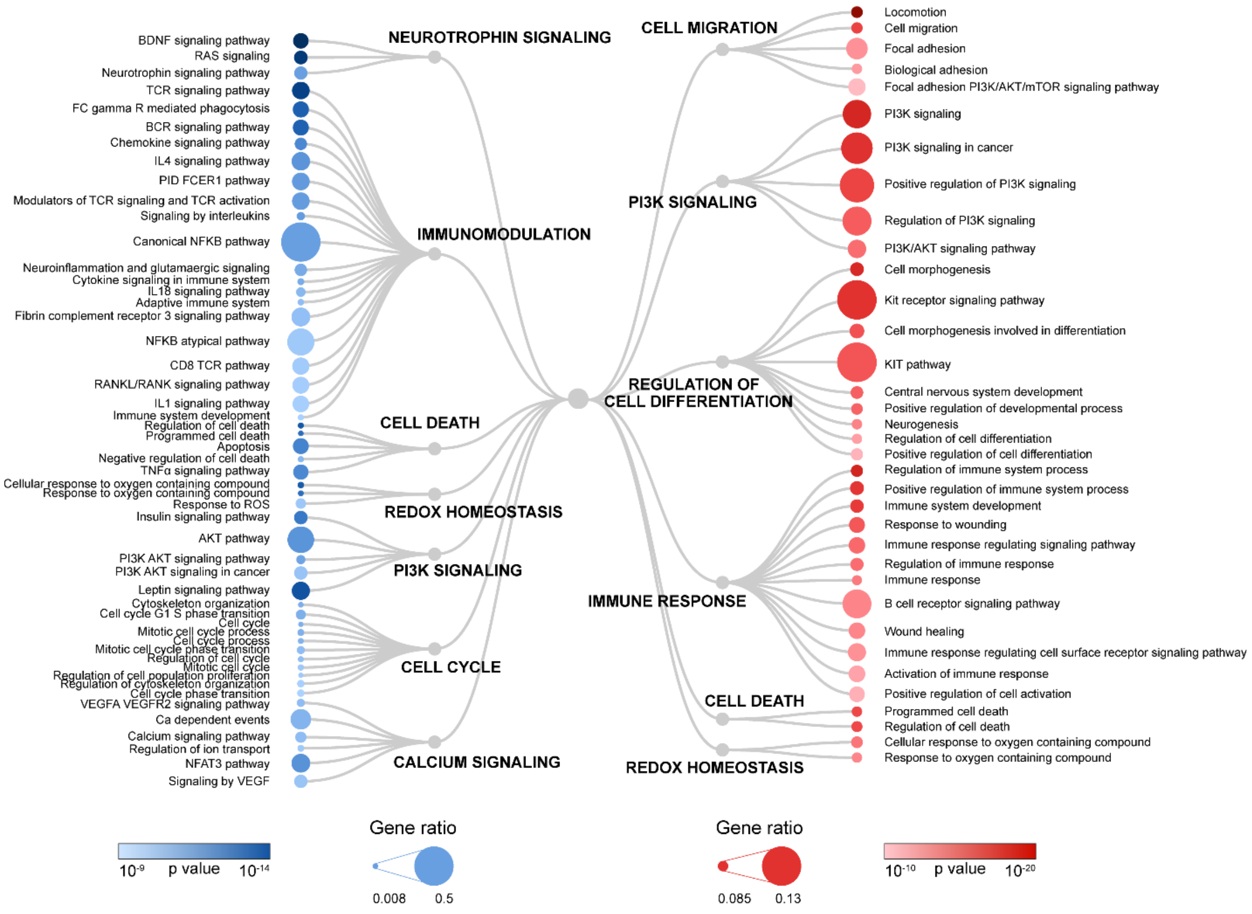

Supplementary Figure S5. Kinase activation-driven enrichments. Diagram reporting the all the significant (FDR<0.05) enrichments displayed by the inferred differentially activated kinases (and their relative differentially phosphorylated target peptides) through GSEA in the C2cp MSigDB collection. Displayed pathway enrichments have been clustered into the indicated "master processes".

Supplementary Figure S6

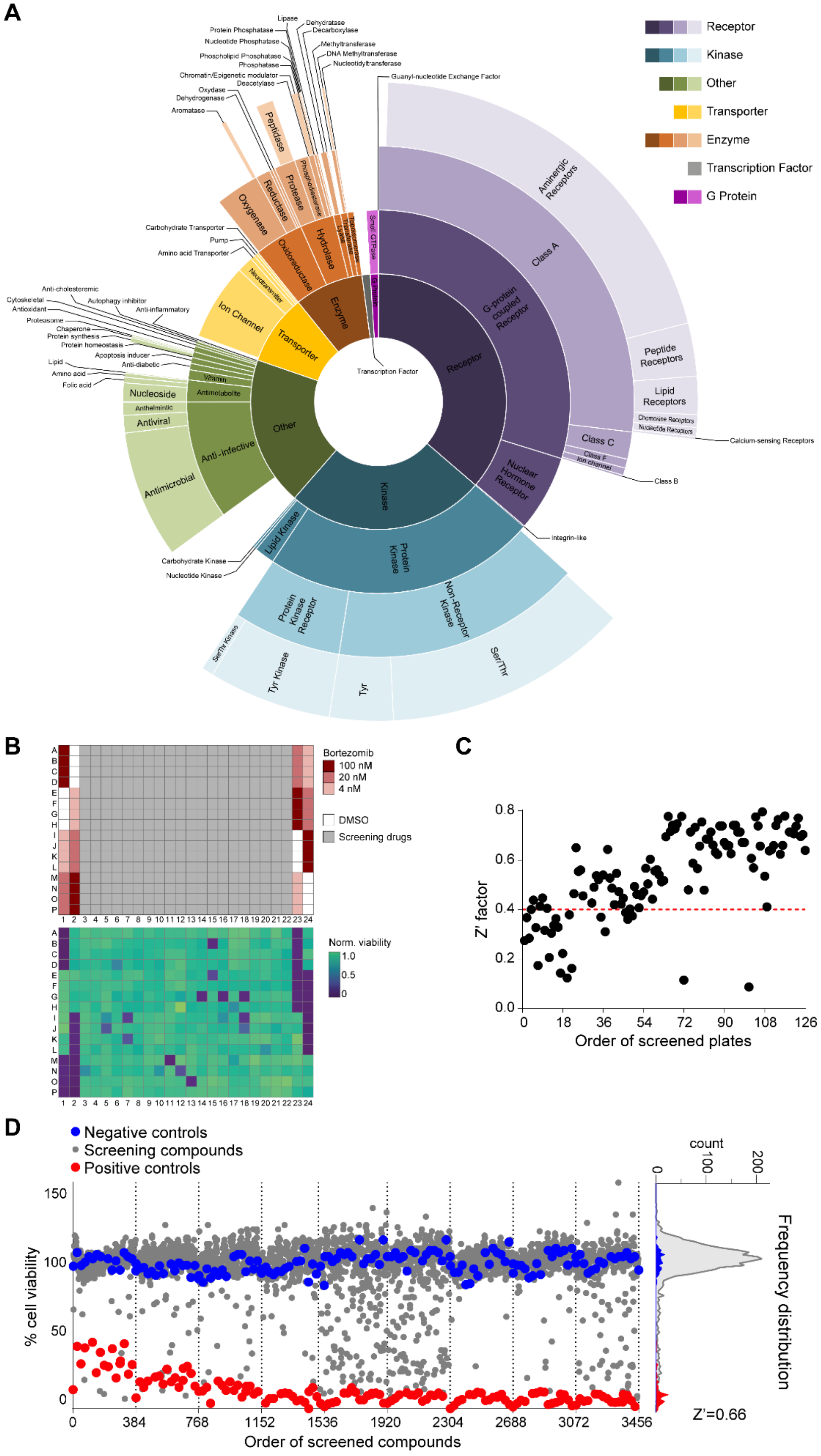

Supplementary Figure S6. Drug library composition and technical screening parameters. (**A**) Distribution of all the compounds composing the drug library used within our HTS approach (n=3533) according to their mechanism of action and pharmacological classification. (**B**) Distribution of negative controls (white, DMSO), library compounds (grey), and positive controls (shades of red, high-dose Bortezomib treatment) within the 384-well plates used for HTS (upper panel) and representative test plate reporting normalized cell viability after treatment (lower panel). (**C**) Dot plot summarizing the distribution of Z' factor values calculated for each test plate screened within our study. (**D**) Representative scatterplot displaying the distribution of library compounds (grey dots), negative controls (blue dots), and positive controls (red dots) according to their arrangement within the different test plates (n=9) and cell viability data (left). Frequency distribution of test compounds and controls is also reported (right).

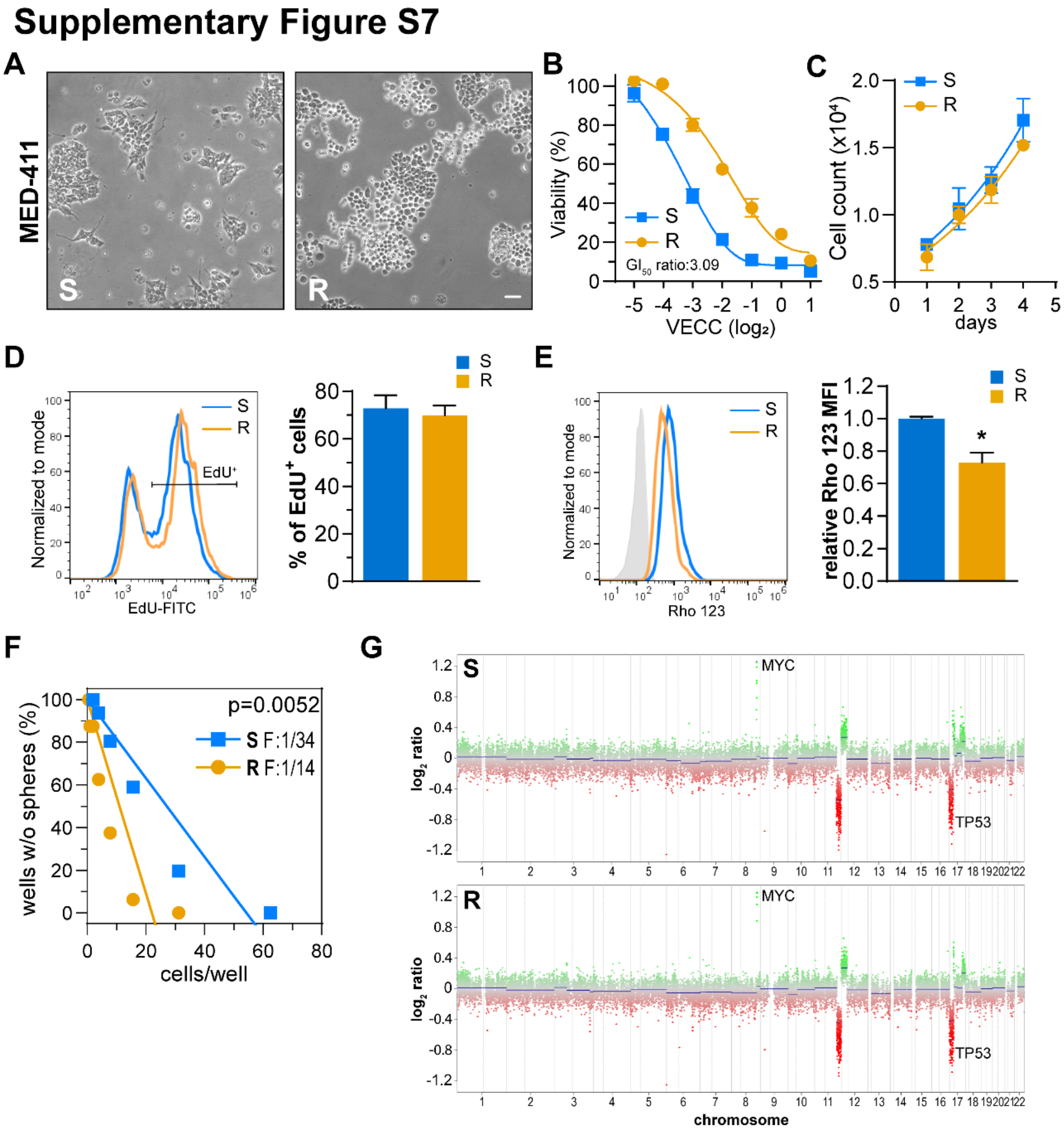

Supplementary Figure S7. Generation and functional characterization of Med-411-S/R primary cultures. (**A**) Representative brightfield images displaying no evident morphological differences between Med-411-S and Med-411-R primary cells. Original magnification 10X; bar: 20µm (**B**) Dose-response curves demonstrating increased resistance to VECC treatment of Med-411-R cells relative to their naïve counterparts (S). (**C**) Curves displaying that both Med-411-S and Med-411-R cellular models possess equivalent growth rates (within 96h) as measured by trypan blue exclusion assays. (**D**) Representative cytofluorimetric plots (left) and relative quantification (right) of Med-411-S/R cell proliferation as measured by the amount of EdU incorporation within 16h (n=5 independent experiments). (**E**) Representative plots (left) and relative quantification (right) of drug efflux potential of Med-411-S/R cells as measured by detection of Rho 123 extrusion by flow cytometry (n=3 independent experiments). Control unstained cells are reported in grey. * $p<0.05$ by paired t test. (**F**) Limiting dilution assays comparing self-renewal potential of Med-411-S/R cells. Initiating cell frequency F of cells is reported. p calculated by extra sum-of-squares F test. (G) Representative analysis of copy number evolution in MED-411-S and R cells.

Supplementary Figure S8

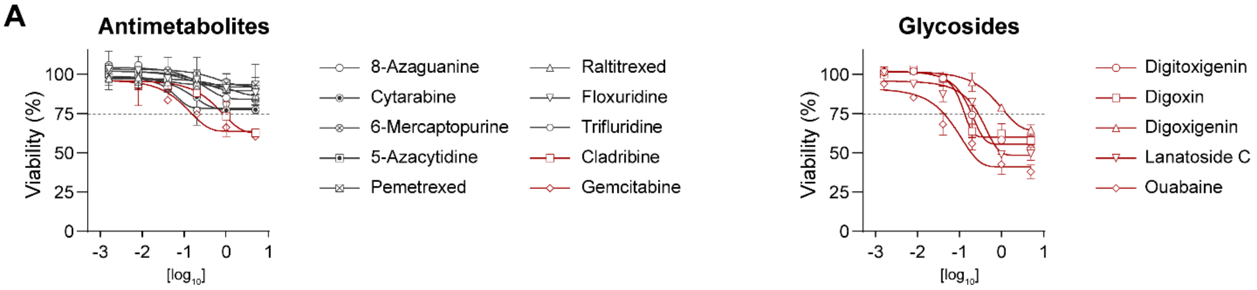

Supplementary Figure S8. Antimetabolite compounds activity in primary human fibroblasts. (**A**) Dose-response curves of antimetabolites (left) and glycosides (right) from Fig. 5A performed in normal human fibroblasts. Drugs reducing normal cell viability below the indicated 75% threshold (dotted line) are highlighted by red curves.

Supplementary Figure S9

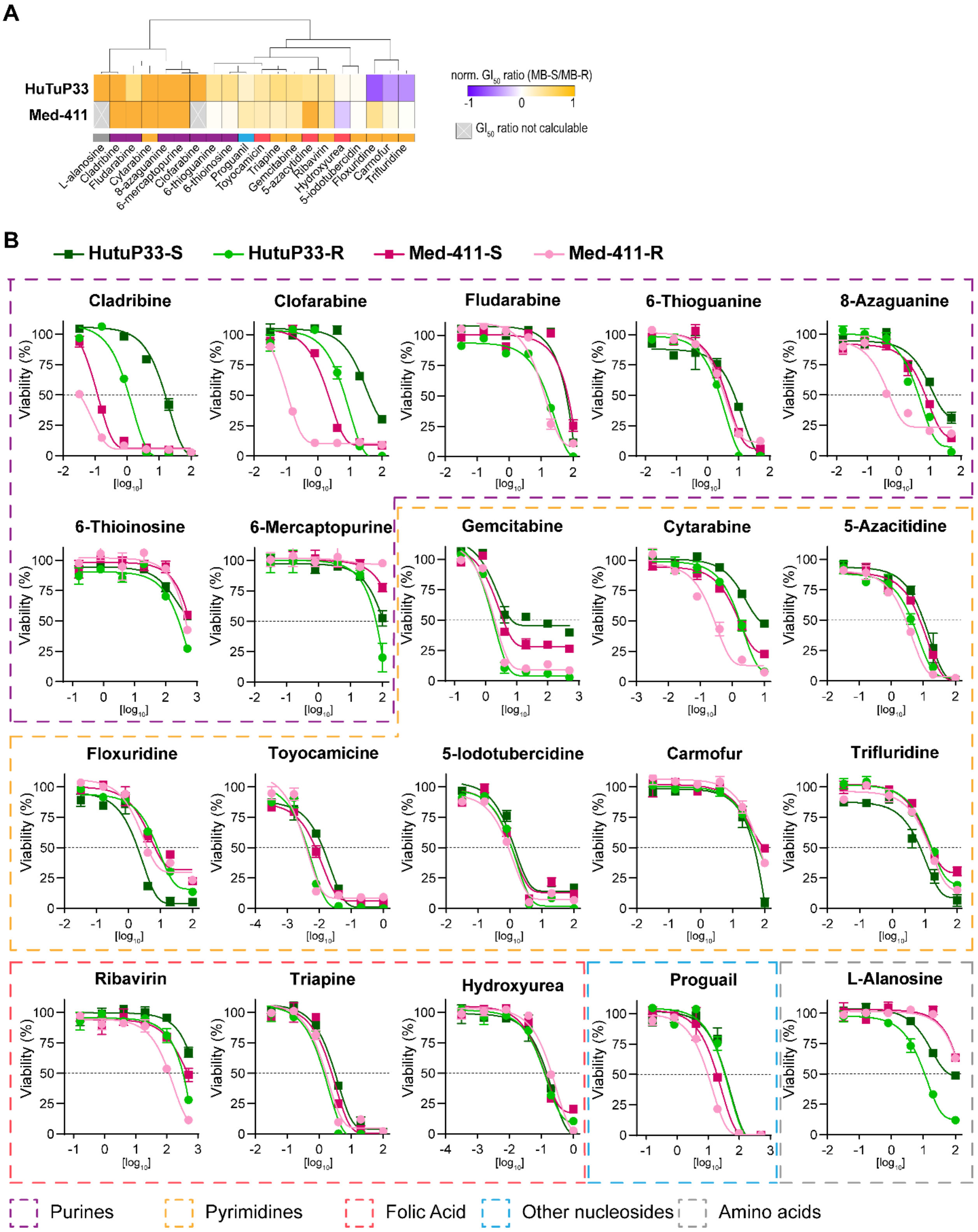

Supplementary Figure S9. Antimetabolite compounds activity in MB primary cultures.
(**A**) Levelplot summarizing the activity of antimetabolite compounds in primary MB cultures by displaying normalized GI_{50} ratios (MB-S GI_{50}/MB-R GI_{50}, when calculable). Indication of compound categorization as in (Figure 6A) is reported. (**B**) Dose-response curves derived from the treatment of HuTuP33-S/R and MED-411-S/R with the indicated antimetabolites. Only dose-response curves from which GI_{50} values were calculable are shown. Antimetabolites are framed into colored dotted boxed based on their categorization as purine analogues (purple), pyrimidine analogues (yellow), folic acid synthesis inhibitors (red), other nucleosides (blue), and amino acids (grey).

Supplementary Figure S10

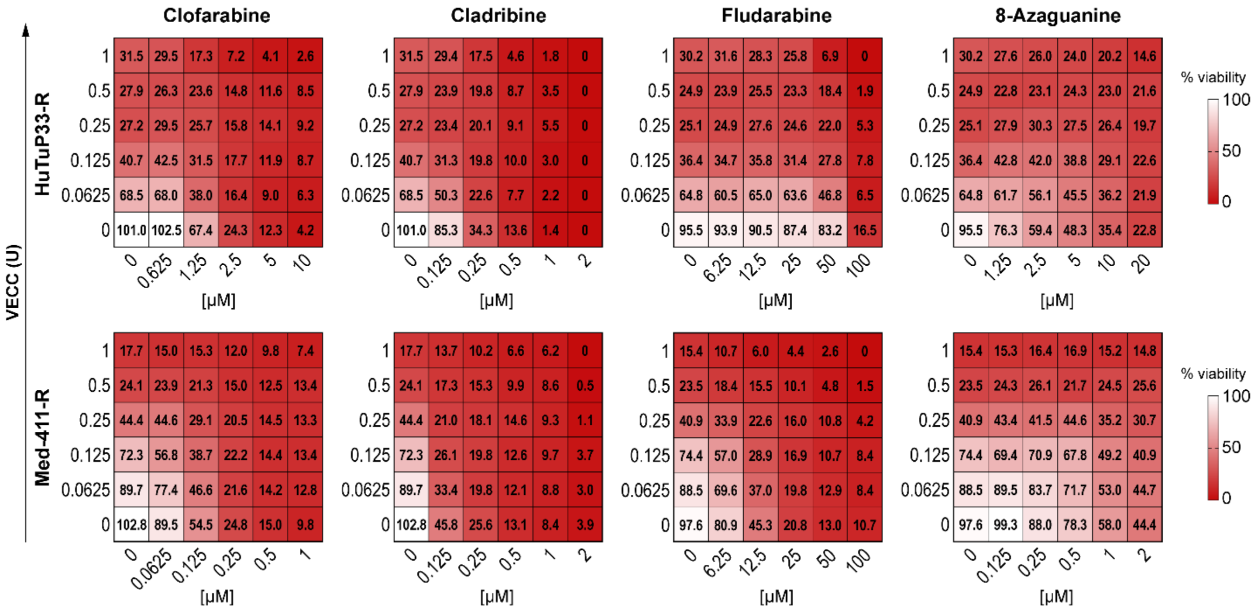

Supplementary Figure S10. Viability matrixes of VECC-antimetabolites combinations. Dose response matrixes displaying viability of HuTuP33-R (top panels) and Med-411 (bottom panels) cells when treated for 72h with scalar doses of VECC combined with scalar doses of selected antimetabolite compounds (as in Fig. 6D) according to a 5x5 (6x6 if considering also control cells administered with the single agents) combination matrix configuration.

REFERENCES OF SUPPLEMENTARY MATERIAL

1. Tyanova S, Temu T, Sinitcyn P, Carlson A, Hein MY, Geiger T, et al. The Perseus computational platform for comprehensive analysis of (prote)omics data. Nat Methods. 2016; 13: 731-40. https://doi.org/10.1038/nmeth.3901.

2. Tusher VG, Tibshirani R, Chu G. Significance analysis of microarrays applied to the ionizing radiation response. Proc Natl Acad Sci U S A. 2001; 98: 5116-21. https://doi.org/10.1073/pnas.091062498.

3. Bussmann L, Hoffer K, von Bargen CM, Droste C, Lange T, Kemmling J, et al. Analyzing tyrosine kinase activity in head and neck cancer by functional kinomics: Identification of hyperactivated Src family kinases as prognostic markers and potential targets. Int J Cancer. 2021; 149: 1166-80. https://doi.org/10.1002/ijc.33606.

4. Krayem M, Aftimos P, Najem A, van den Hooven T, van den Berg A, Hovestad-Bijl L, et al. Kinome Profiling to Predict Sensitivity to MAPK Inhibition in Melanoma and to Provide New Insights into Intrinsic and Acquired Mechanism of Resistance. Cancers (Basel). 2020; 12. https://doi.org/10.3390/cancers12020512.

5. Hafner M, Niepel M, Chung M, Sorger PK. Growth rate inhibition metrics correct for confounders in measuring sensitivity to cancer drugs. Nat Methods. 2016; 13: 521-7. https://doi.org/10.1038/nmeth.3853.

6. Zheng S, Wang W, Aldahdooh J, Malyutina A, Shadbahr T, Tanoli Z, et al. SynergyFinder Plus: Toward Better Interpretation and Annotation of Drug Combination Screening Datasets. Genomics Proteomics Bioinformatics. 2022. https://doi.org/10.1016/j.gpb.2022.01.004.

7. Hovestadt V, Remke M, Kool M, Pietsch T, Northcott PA, Fischer R, et al. Robust molecular subgrouping and copy-number profiling of medulloblastoma from small amounts of archival tumour material using high-density DNA methylation arrays. Acta Neuropathol. 2013; 125: 913-6. https://doi.org/10.1007/s00401-013-1126-5.

8. Cavalli FMG, Remke M, Rampasek L, Peacock J, Shih DJH, Luu B, et al. Intertumoral Heterogeneity within Medulloblastoma Subgroups. Cancer Cell. 2017; 31: 737-54 e6. https://doi.org/10.1016/j.ccell.2017.05.005.
