# Supplementary material for: Molecular and functional profiling of chemotolerant cells unveils nucleoside metabolism-dependent vulnerabilities in medulloblastoma
Source: Acta Neuropathol Commun. 2023 Nov 17;11:183. doi: 10.1186/s40478-023-01679-7 (PMC10655385; doi:10.1186/s40478-023-01679-7)
Supplement: Supplementary file 4 — Additional file 4. Supplementary Table S3. [file 40478_2023_1679_MOESM4_ESM.docx]

Supplementary Table S3. Running parameters used for MaxQuant

| General |  |
| --- | --- |
| MaxQuant version | 1.6.10.43 |
| LC-MS run type | Standard |
| Multiplicity | 1 |
| Labels | - |
|  |  |
| Digestion |  |
| Enzyme mode | Specific |
| Enzyme | Trypsin/P |
| Max. missed cleavages | 2 |
| Separate enzyme for first search | True |
|  |  |
| Modifications |  |
| Fixed modifications | Carbamidomethyl (C) |
| Variable modifications | Oxidation (M);Acetyl (N-term) |
| Max. number of modifications per peptide | 5 |
| Separate variable modifications for first search | True |
|  |  |
|  |  |
| Sequences |  |
| Fasta file | UniProtKB/SwissProt *Homo Sapiens* (reviewed=YES) |
| Include contaminants | True |
| Decoy mode | revert |
| Min. peptide length | 7 |
| Max. peptide mass | 4600 Da |
| Min. peptide length for unspecific search | 8 |
| Max. peptide length for unspecific search | 25 |
| Variation mode | None |
|  |  |
| Identification |  |
| PSM FDR | 0,01 |
| Protein FDR | 0,01 |
| Site decoy fraction | 0,01 |
| Min. peptides | 1 |
| Min. razor + unqiue peptides | 1 |
| Min. unique peptides | 0 |
| Min. score for unmodified peptides | 0 |
| Min. score for modified peptides | 40 |
| Min. delta score for unmodified peptides | 0 |
| Min. delta score for modified peptides | 6 |
| Main search max. combinations | 200 |
| Base FDR calculations on delta score | False |
| Razor protein FDR | True |
| Second peptides | True |
| Match between runs | True |
| Match time window (min) | 0.7 |
| Match ion mobility window | 0.05 |
| Alignment time window (min) | 20 |
| Alignment ion mobility | 1 |
| Match unidentified features | False |
| Labeled amino acid filtering | True |
| Dependent peptides | False |
|  |  |
| Quantification |  |
| Label-free quantification | True |
| LFQ min. ratio count | 1 |
| Fast LFQ | False |
| Skip normalization | False |
| Label min. ratio count | 2 |
| Use only unmodified peptides and | True |
| Modifications included in protein quantification | Oxidation (M);Acetyl (Protein N-term) |
| Peptides used for protein quantification | Unique |
| Discard unmodified counterpart peptides | True |
| Advanced ratio estimation | True |
| Separate LFQ in parameter groups | False |
| Stabilize large LFQ ratios | True |
| Require MS/MS for LFQ comparisons | True |
| iBAQ | True |
| iBAQ log fit | True |
| Advanced site intensities | True |
| Re-quantify | False |
|  |  |
| Instrument |  |
| Instrument type | Orbitrap |
| First search peptide tolerance | 20 ppm |
| Main search peptide tolerance | 4.5 ppm |
| Individual peptide mass tolerance | True |
| Isotope match tolerance | 2 ppm |
| Centroid match tolerence | 8 ppm |
| Centroid half width | 35 ppm |
| Time valley factor | 1.4 |
| Isotope valley factor | 1.2 |
| Isotope time correlation | 0.6 |
| Theoretical isotope correlation | 0.6 |
| Recalibration unit | ppm |
| Use MS1 centroids | False |
| Use MS2 centroids | False |
| Intensity dependent calibration | False |
| Min. peak length | 2 |
| Max. charge | 7 |
| Min score for recalibration | 70 |
| Cut peaks | True |
| Gap scans | 1 |
| Advanced peak splitting | False |
| Intensity threshold | 0 |
| Intensity determination | Value at maximum |
